# Supplementary material for: CTCF mutation at R567 causes developmental disorders via 3D genome rearrangement and abnormal neurodevelopment
Source: Nat Commun. 2024 Jul 1;15:5524. doi: 10.1038/s41467-024-49684-1 (PMC11217373; doi:10.1038/s41467-024-49684-1)

SUPPLEMENTARY INFORMATION

CTCF mutation at R567 causes developmental disorders by 3D genome rearrangement and abnormal neurodevelopment

Supplementary information includes:

Supplementary Figure 1-10.

Supplementary Table 1. List of sgRNA and ssODN sequences used for genome editing.

Supplementary Table 2. List of primers used for genotyping, Hi-C and QHR-4C experiments.

Supplementary Table 3. List of primers used for RT-qPCR experiments.

Supplementary Table 4. List of primers and probes used for EMSA/GST-pull down assays.

Supplementary Table 5. List of primers used for ChIP-qPCR experiments.

Supplementary Table 6. List of antibodies used in this study.

Uncropped gel and blots.

Supplementary Fig. 1

a

Genotypes of *Ctcf* mutant mice

stage	$Ctcf^{+/+}$	$Ctcf^{+/R567W}$	$Ctcf^{R567W/R567W}$	Total	Litters	P
E14.5	8	22	8	38	4	0.6227
E18.5	67	142	46	255	29	0.0341
P0	40	50	*32	122	14	0.0814
P1	36	43	0	79	14	< 0.0001

* Died within 30 minutes after birth

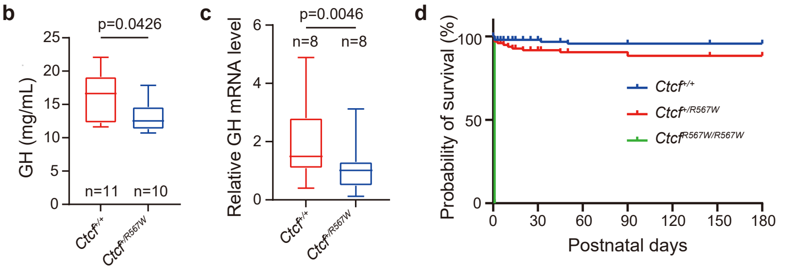

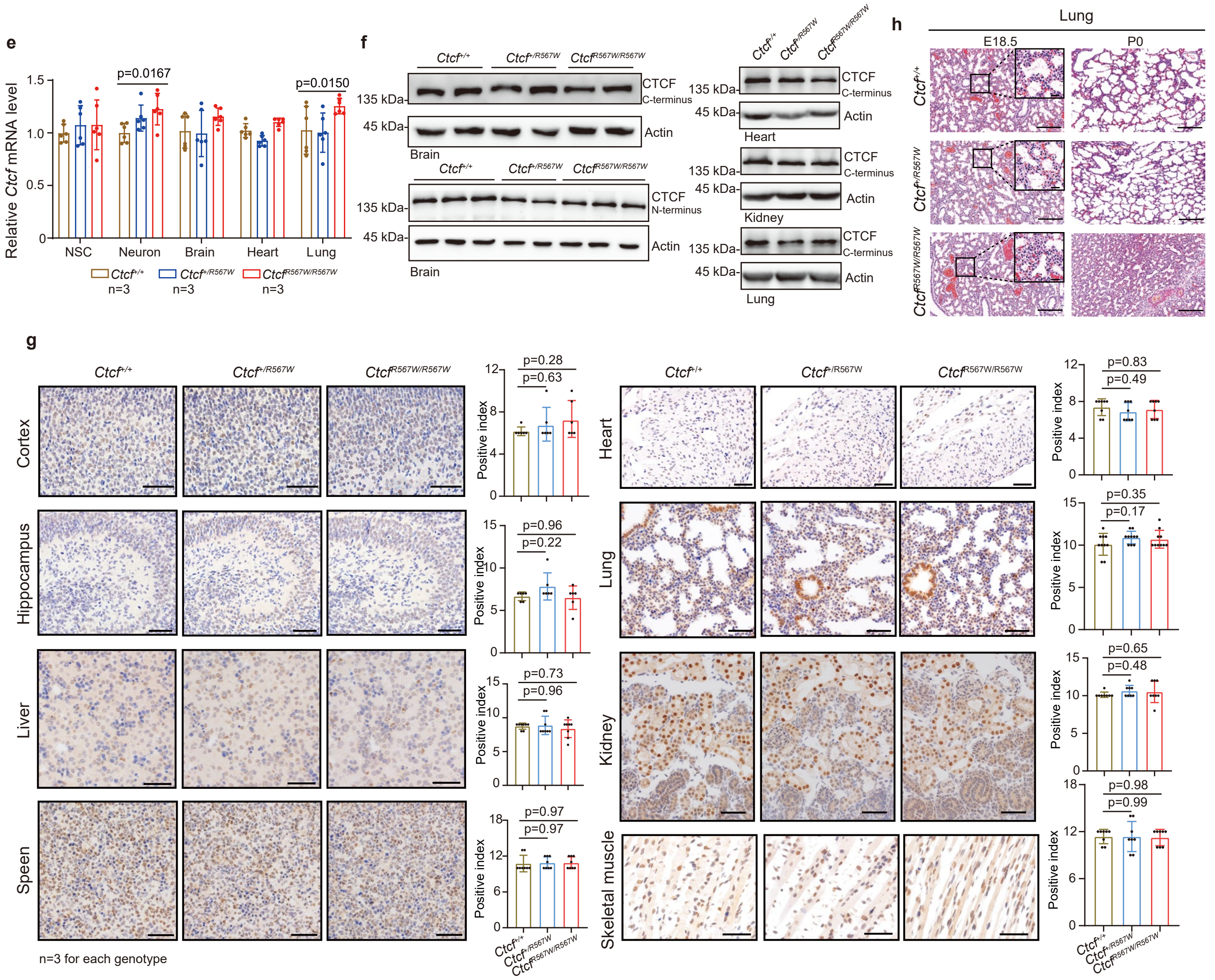

Supplementary Figure 1. Phenotype of CTCF^{R567W} mutant mice. (**a**) Genotype frequency of progeny from the mating of *Ctcf* mutant mice. Asterisks* indicate the number of mice that died within 30 min after birth. (**b**) Box and whisker plots showing the ELISA results of the growth hormone (GH) content (95% confidence interval) in blood from 3-week-old *Ctcf*$^{+/+}$ and *Ctcf*$^{+/R567W}$ mice (n = 11 for *Ctcf*$^{+/+}$, n = 10 for *Ctcf*$^{+/R567W}$). (**c**) Box and whisker plots showing the RT-qPCR analysis of *GH* mRNA levels (95% confidence interval) in pituitaries isolated from 3-week-old *Ctcf*$^{+/+}$ and *Ctcf*$^{+/R567W}$ mice (n = 8 for each genotype). (**d**) Survival curves of *Ctcf*$^{+/+}$, *Ctcf*$^{+/R567W}$ and *Ctcf*$^{R567W/R567W}$ mice (n = 83 for *Ctcf*$^{+/+}$, n = 137 for *Ctcf*$^{+/R567W}$, n = 52 for *Ctcf*$^{R567W/R567W}$). (**e**) Quantitative PCR analysis of *Ctcf* mRNA levels in different tissues isolated from E18.5 embryos of *Ctcf*$^{+/+}$, *Ctcf*$^{+/R567W}$ and *Ctcf*$^{R567W/R567W}$ (n = 3 for each genotype). (**f**) Western blot analysis of CTCF protein levels in different tissues isolated from E18.5 embryos of *Ctcf*$^{+/+}$, *Ctcf*$^{+/R567W}$ and *Ctcf*$^{R567W/R567W}$ mice. (**g**) Immunohistochemistry images (left) and quantitative analysis results (right) showing CTCF expression and distribution in various tissues, including the brain (cortex and hippocampus), heart, lung, liver, kidney, spleen and skeletal muscle of E18.5 mice (n = 3 for each genotype; at least 200 cells were recorded in each field from approximately 10 fields). Scale bars, 50 μm. (**h**) Representative H&E staining images of paraffin lung sections from *Ctcf*$^{+/+}$, *Ctcf*$^{+/R567W}$ and *Ctcf*$^{R567W/R567W}$ mice at E18.5 (left) or P0 (right). Scale bars, 200 μm. Quantitative data are presented as the mean ± SD. The *p*-values obtained by Chi-Squared test (**a**), two-tailed unpaired *t*-test (**b** and **c**) and one-way ANOVA with Dunnett's multiple comparisons test (**e** and **g**) are indicated. For **c**, and **e-h**, these experiments were repeated independently three times with similar results. All Box and whisker plots show lower and upper quartiles (box limits), median (centre line) and minimum to maximum values (whiskers). Source data are provided as a Source Data file.

Supplementary Fig. 2

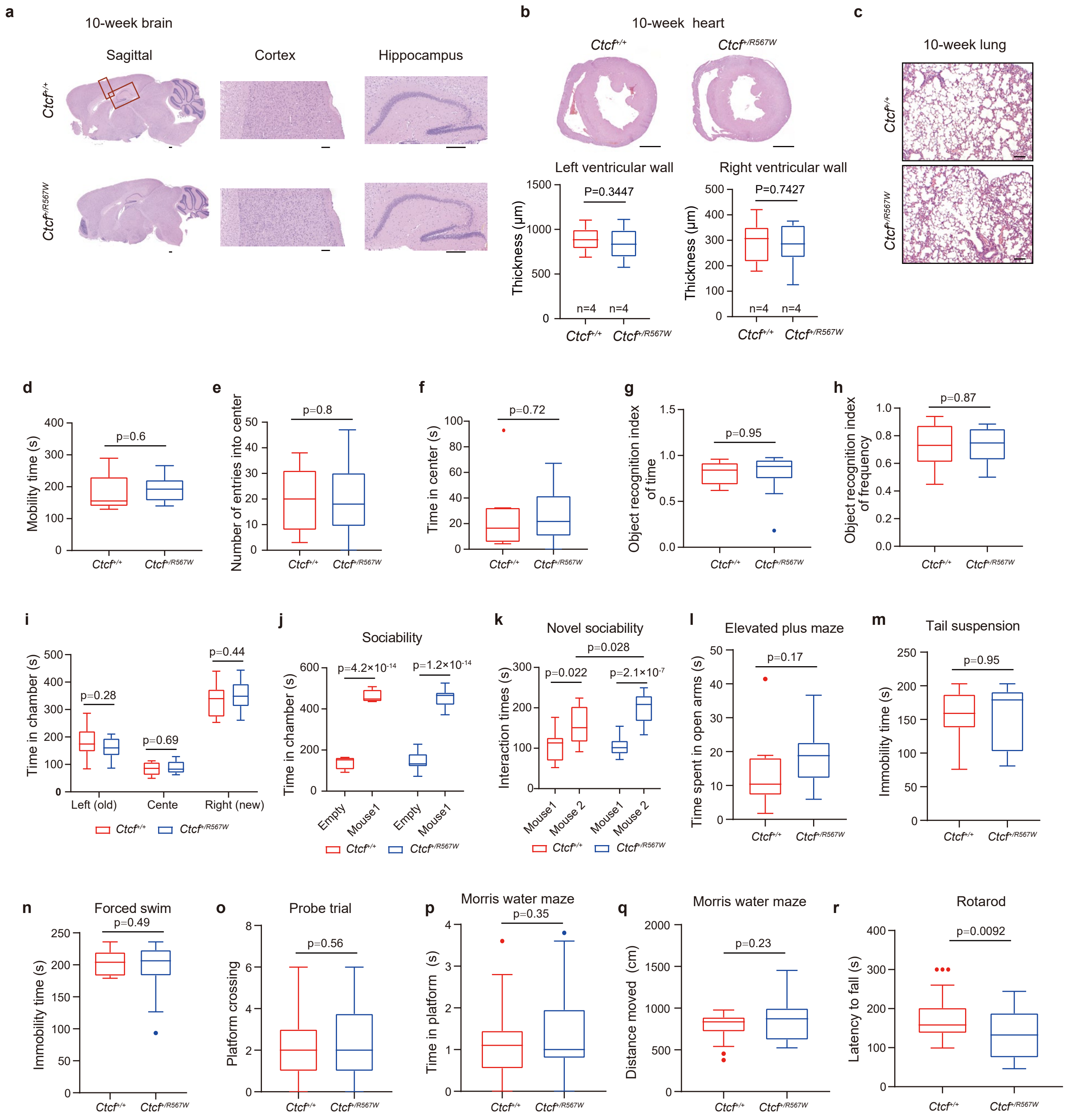

d-r: n=11 for *Ctcf*+/+ and n=12 for *Ctcf*+/R567W

Supplementary Figure 2. Tissue imaging and behavioral tests for *Ctcf*$^{+/+}$ and *Ctcf*$^{+/R567W}$ mice. (**a**) H&E images of sagittal brain sections from 10-week-old *Ctcf*$^{+/+}$ and *Ctcf*$^{+/R567W}$ mice. Enlarged cortex and hippocampus views are on the right. Scale bars, 200 µm. (**b**) (Top) H&E images of heart cross sections from 10-week-old *Ctcf*$^{+/+}$ and *Ctcf*$^{+/R567W}$ mice. Scale bars, 1 mm. (Bottom) Box and whisker plots showing the quantification of left and right ventricular walls in the top images. Each section was randomly measured three times (n = 4 for each genotype). (**c**) H&E images of the lungs of 10-week-old *Ctcf*$^{+/+}$ and *Ctcf*$^{+/R567W}$ mice. Scale bars, 200 µm. (**d-f**) Open-field test results: mobility time (**d**), number of entries into the center (**e**) and time in the center (**f**). (**g**, **h**) Novel object recognition test results: object recognition index of time (**g**) and frequency (**h**). Object recognition index of time (frequency) = the time (frequency) of exploring the new object/total time (frequency) of exploring the new and old objects. (**i**) Quantification of time spent in each chamber in the three-chamber sociability test and novel sociability test. (**j**, **k**) Time spent in the chamber (**j**) and interaction times (**k**) in the three-chamber sociability test and novel sociability test. (**l**) The time spent in open arms from the elevated plus maze test. (**m**, **n**) Immobility time in tail suspension (**m**) and forced swim test (**n**). (**o**) Latency to find the hidden platform in Morris water maze experiments. (**p**, **q**) Time in platform (**p**) and distance moved (**q**) during the probe trial. (**r**) Latency to fall during the rotarod test on day 4. Data are presented as the mean ± SD. The *p*-values obtained by two-tailed unpaired *t*-test are indicated. n = 11 for *Ctcf*$^{+/+}$ and n = 12 for *Ctcf*$^{+/R567W}$ for all behavioral tests (**d-r**). For **a-c**, these experiments were repeated three times independently with similar results. All Box and whisker plots show lower and upper quartiles (box limits), median (centre line) and minimum to maximum values (whiskers), representing 95% confidence intervals. Source data are provided as a Source Data file.

Supplementary Fig. 3

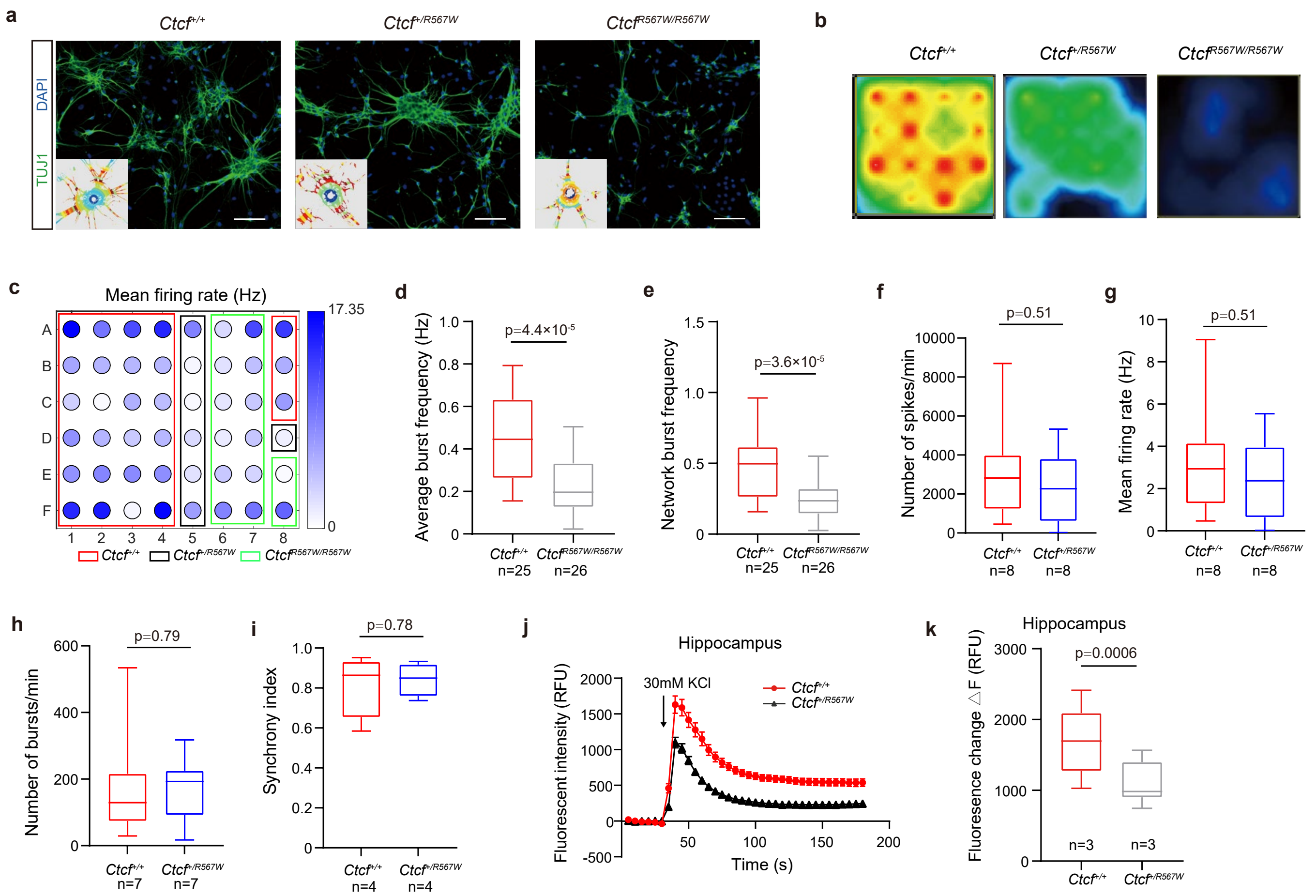

Supplementary Figure 3. Effects of the CTCF^{R567W} mutation on neural electrical activity. (**a**) Fluorescence images of TUJ1 (green) immunostaining in neural stem cell masses over 2 weeks of culture from E18.5 mouse forebrains. The intersection mask (bottom left) shows the density of neural protrusions. Nuclei were stained with DAPI (blue). Scale bars, 100 µm. (**b**) Heatmap showing a snapshot of the electrical activity of the same array of cultured neurons in *Ctcf*$^{+/+}$, *Ctcf*$^{+/R567W}$ and *Ctcf*$^{R567W/R567W}$ mice in the MEA well. (**c**) Plate map showing the mean firing rate of the MEA experiment on day 14. Different colored rectangular boxes represent the culture wells of different genotypes. (**d**, **e**) Box and whisker plots showing the quantification of the MEA metrics (95% confidence interval) of *Ctcf*$^{+/+}$ and *Ctcf*$^{R567W/R567W}$ cultured neurons. Statistics of electrical activity for 15 min, including the average burst frequency (**d**) and the network burst frequency (**e**) (n = 4 for *Ctcf*$^{+/+}$, n = 2 for *Ctcf*$^{R567W/R567W}$; the n of cultured wells recorded are indicated in the graph). (**f-i**) Box and whisker plots showing the quantification of the MEA metrics (95% confidence interval) of *Ctcf*$^{+/+}$ and *Ctcf*$^{+/R567W}$ cultured neurons. Statistics of electrical activity for 15 min, including the number of spikes per min (**f**), the mean firing rate (**g**), the number of bursts per min (**h**) and the synchrony index (**i**) (n = 3 for each genotype, the n of cultured wells recorded are indicated in the graph). (**j**, **k**) Calcium influx after the depolarization of cultured hippocampal neurons in *Ctcf*$^{+/+}$ and *Ctcf*$^{R567W/R567W}$ mice (n = 3 for each genotype) (represented by 95% confidence interval; RFU: relative fluorescence units). Quantitative data are presented as the mean ± SD. The *p*-values obtained by two-tailed unpaired *t*-test are indicated. For **a**, and **d-k**, these experiments were repeated three times independently with similar results. All Box and whisker plots show lower and upper quartiles (box limits), median (centre line) and minimum to maximum values (whiskers). Source data are provided as a Source Data file.

Supplementary Fig. 4

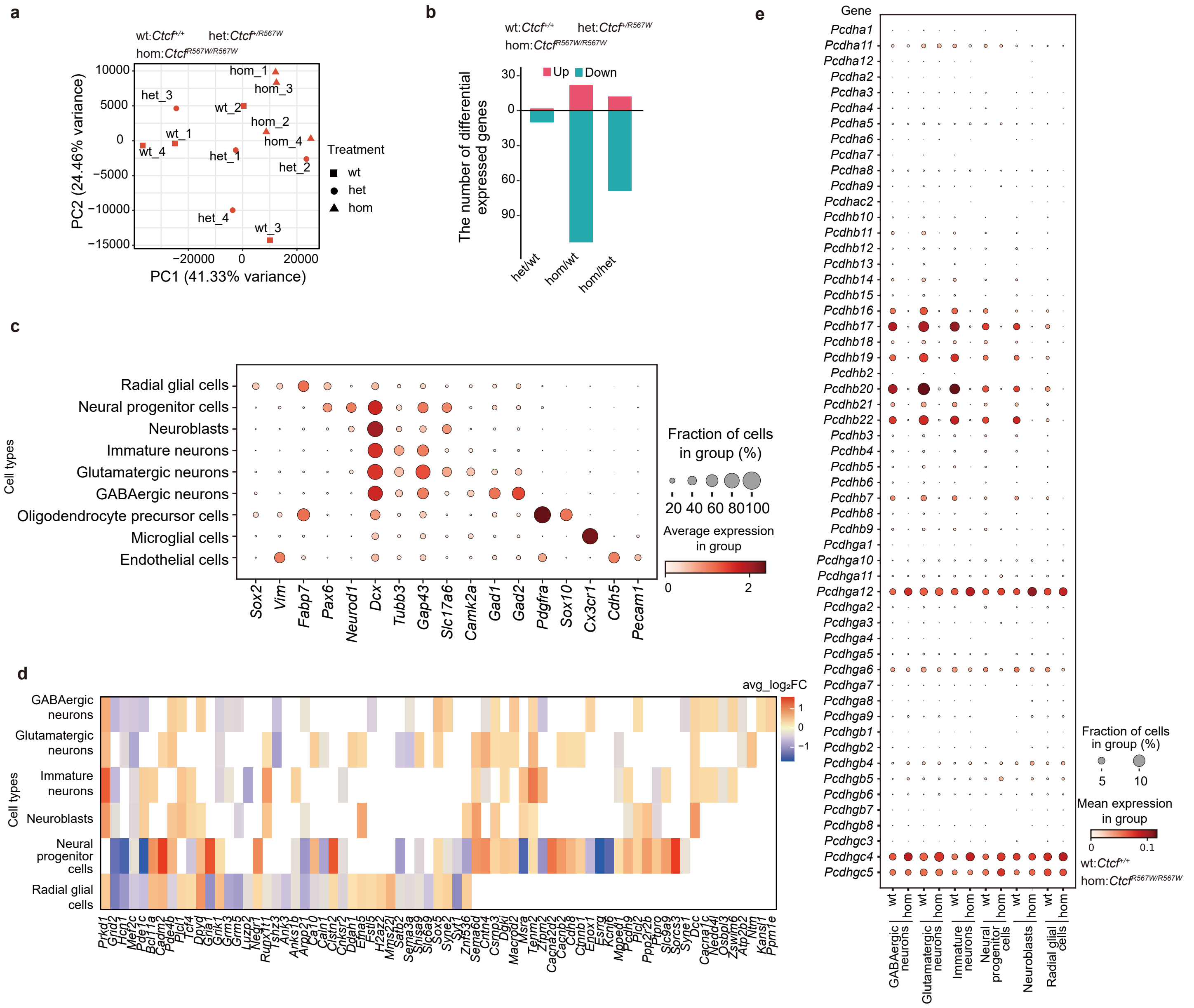

Supplementary Figure 4. Additional information for bulk RNA-seq from brains and snRNA-seq from Cortices. (**a**) PCA of RNA-seq data from the whole brains of E18.5 *Ctcf*$^{+/+}$, *Ctcf*$^{+/R567W}$ and *Ctcf*$^{R567W/R567W}$ mice (n = 4 for each genotype). (**b**) Number of DEGs in whole brains among different genotypes from RNA-seq. (**c**) Dot plot showing the relative expression of selected marker genes for each cell type in the cortex from snRNA-seq. (**d**) Heatmap showing the DEGs from each cell type of E18.5 cortex snRNA-seq overlapping with ASD-related GWAS risk genes. (**e**) Dot plot showing *cPcdh* gene expression across cell types in snRNA-seq data of E18.5 *Ctcf*$^{+/+}$ and *Ctcf*$^{R567W/R567W}$ cortex samples. The size of each circle reflects the percentage of cells within a specific cell type that expressed the given gene. The color indicates the average expression level of that gene within the specific cell population.

Supplementary Fig. 5

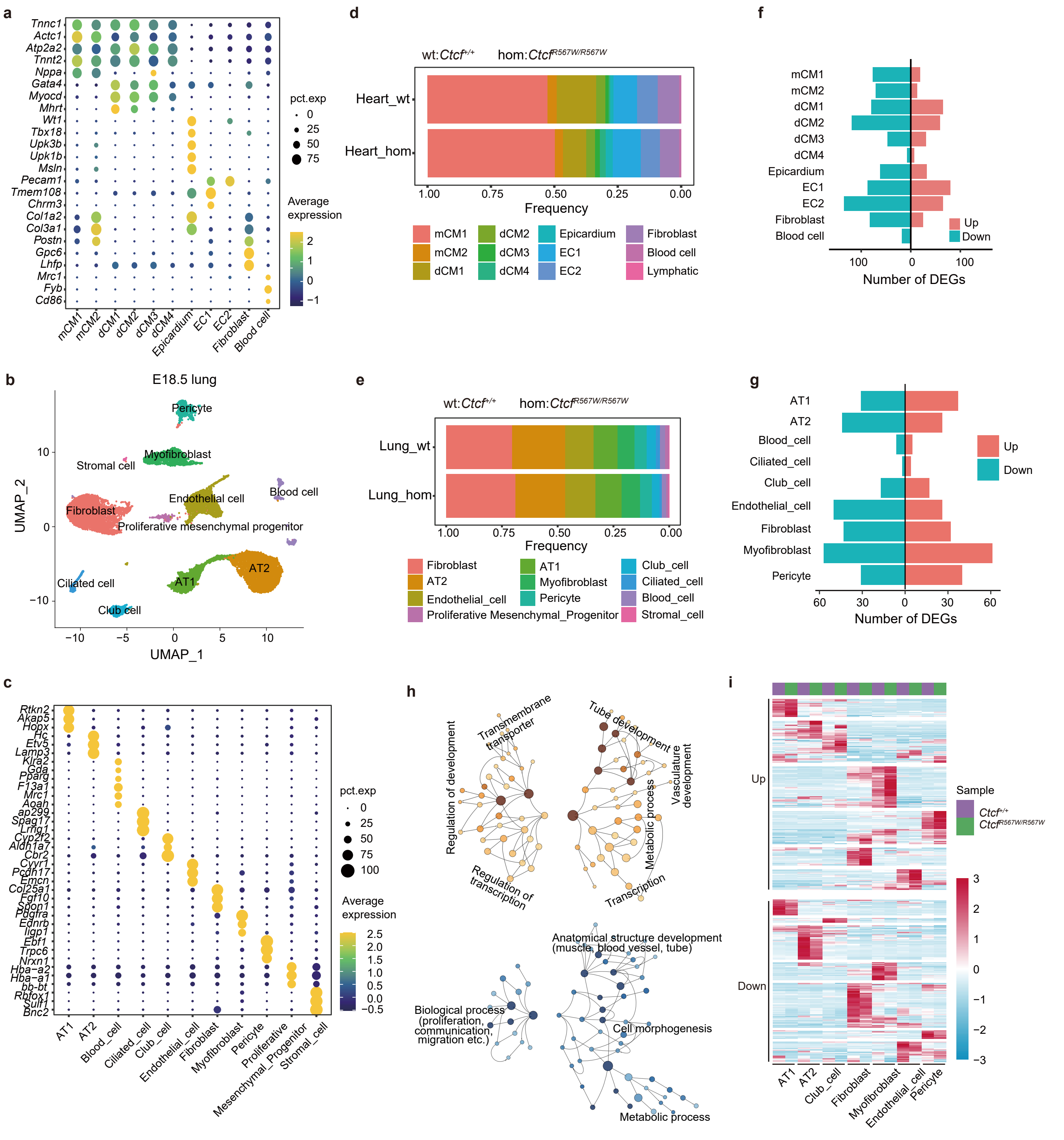

Supplementary Figure 5. Additional information for snRNA-seq from heart and lung tissues. (**a**) Dot plot showing the relative expression of selected marker genes for each cell type in the heart from snRNA-seq. (**b**) UMAP visualization of snRNA-seq data and cell clustering results of lung samples from E18.5 *Ctcf*$^{+/+}$ and *Ctcf*$^{R567W/R567W}$ mice. AT1, alveolar type 1 cells; AT2, alveolar type 1 cells. (**c**) Dot plot showing the relative expression of selected marker genes for each cell type in the lungs from snRNA-seq. (**d, e**) Proportions of different cell types in heart (**d**) and lung (**e**) snRNA-seq of *Ctcf*$^{+/+}$ and *Ctcf*$^{R567W/R567W}$ mice. (**f, g**) The number of DEGs for each cell type in the heart (**f**) and lung (**g**) samples of E18.5 *Ctcf*$^{+/+}$ and *Ctcf*$^{R567W/R567W}$ mice from snRNA-seq data. (**h**) GO networks of DEGs from lung snRNA-seq data. The significantly up- or downregulated genes of each cell type between *Ctcf*$^{+/+}$ and *Ctcf*$^{R567W/R567W}$ lung samples were merged. (**i**) Heatmap showing the average expression of DEGs from each cell type in lung samples between *Ctcf*$^{+/+}$ and *Ctcf*$^{R567W/R567W}$ mice.

Supplementary Fig. 6

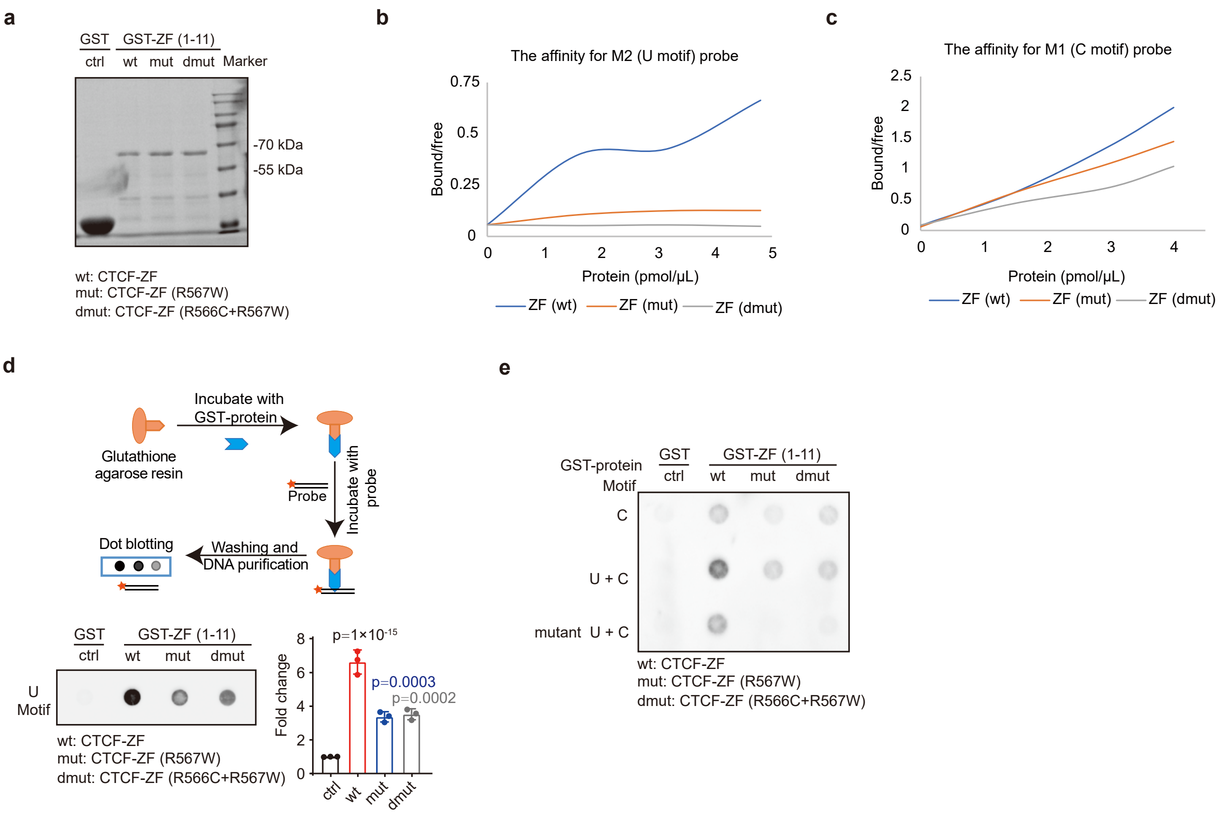

Supplementary Figure 6. Reduced binding affinity of CTCF^{R567W} for CTCF motifs *in vitro*. (**a**) Coomassie brilliant blue staining showing the purified GST, GST-CTCF-ZF-wt, GST-CTCF-ZF-mut (R567W) and GST-CTCF-ZF-dmut (R566C+R567W) proteins electrophoresed on SDS-PAGE gels. (**b, c**) The binding affinity of purified GST-proteins to two motif probes (M2, U motif in **b**; M1, C motif in **c**) was quantified. Binding curves were generated depicting the ratio of bound to free probes for each motif with increasing protein concentration, based on quantification of EMSA results performed in triplicate. (**d**) Flow chart of the GST pull-down assay (top). Dot blot assay showing the enrichment of the biotin-labeled U motif probe by purified GST-fusion proteins (bottom left). Quantification of the dot blot results (bottom right). (**e**) GST pull-down combined with dot blot assay showing the enrichment of biotin-labeled C, U+C, and mutant U+C motif probes by purified GST-fusion proteins. Quantitative data are presented as the mean ± SD. The *p*-values from one-way ANOVA with Dunnett's multiple comparisons test are indicated. These experiments were repeated at least twice independently with similar results. Source data are provided as a Source Data file.

Supplementary Fig. 7

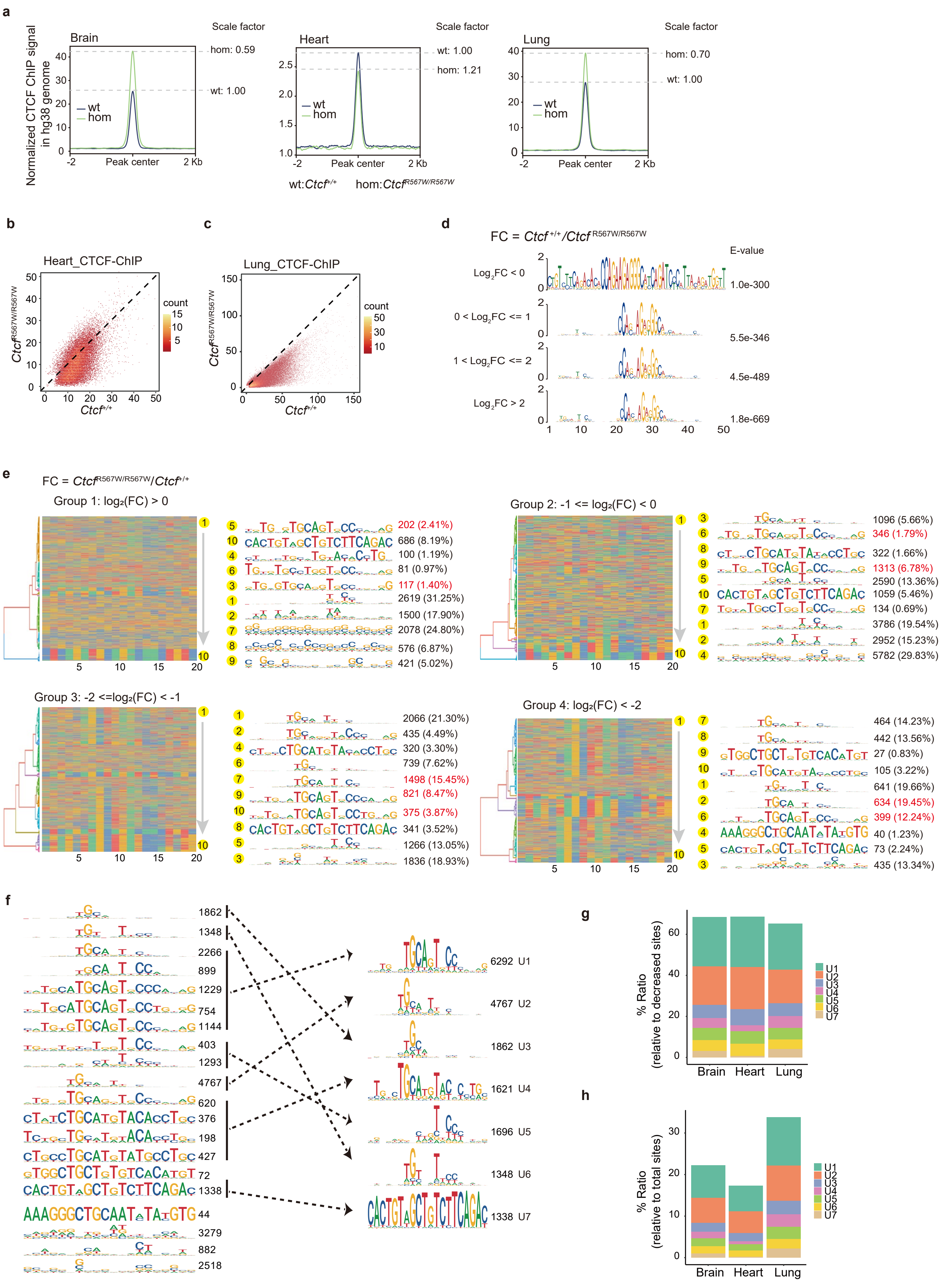

Supplementary Figure 7. Reduced affinity of CTCF^{R567W} for CTCF motifs *in vivo*. (**a**) Profiles showing the average CTCF binding strength in HEK293T cells mixed in different tissue samples of *Ctcf*$^{+/+}$ (black line) or *Ctcf*$^{R567W/R567W}$ (green line) mice. (**b**, **c**) Comparison of normalized CTCF binding strength detected by ChIP-seq of heart (**b**) and lung (**c**) tissues of *Ctcf*$^{+/+}$ and *Ctcf*$^{R567W/R567W}$ mice. (**d**) DNA motif analysis of CTCF binding sites for four groups (categorized by the degree of downregulation of CTCF binding strength after CTCF^{R567W} mutation) in brain tissues. (**e**) Hierarchical clustering of CTCF binding motifs. CTCF sites were divided into 4 groups based on binding strength changes after CTCF mutation. Upstream 20 bp sequences of each site were extracted and clustered. Each group was further clustered into 10 subclusters ordered from top to bottom. Sequence logos were generated for each subcluster and reordered based on similarity. The number of sequences in each subcluster is shown on the right. The red subclusters show sequence logos similar to the canonical U motif. (**f**) U motifs at downregulated CTCF sites. Upstream 20 bp sequences from all downregulated sites ($\log_2$(fold change of *Ctcf*$^{+/+}$/*Ctcf*$^{R567W/R567W}$) > 1) in brain, heart and lungs were combined and clustered into 20 subclusters. Sequence logos were generated for each subcluster. Subclusters with similar logos were combined, resulting in 7 motifs sharing features with the canonical upstream (U) motif. (**g**) Bar plots showing the ratio of CTCF sites containing different kinds of U motifs to decreased CTCF sites after CTCF^{R567W} mutation in each tissue. (**h**) Bar plots showing the ratio of CTCF sites containing different kinds of U motifs to total CTCF sites in each tissue.

Supplementary Fig. 8

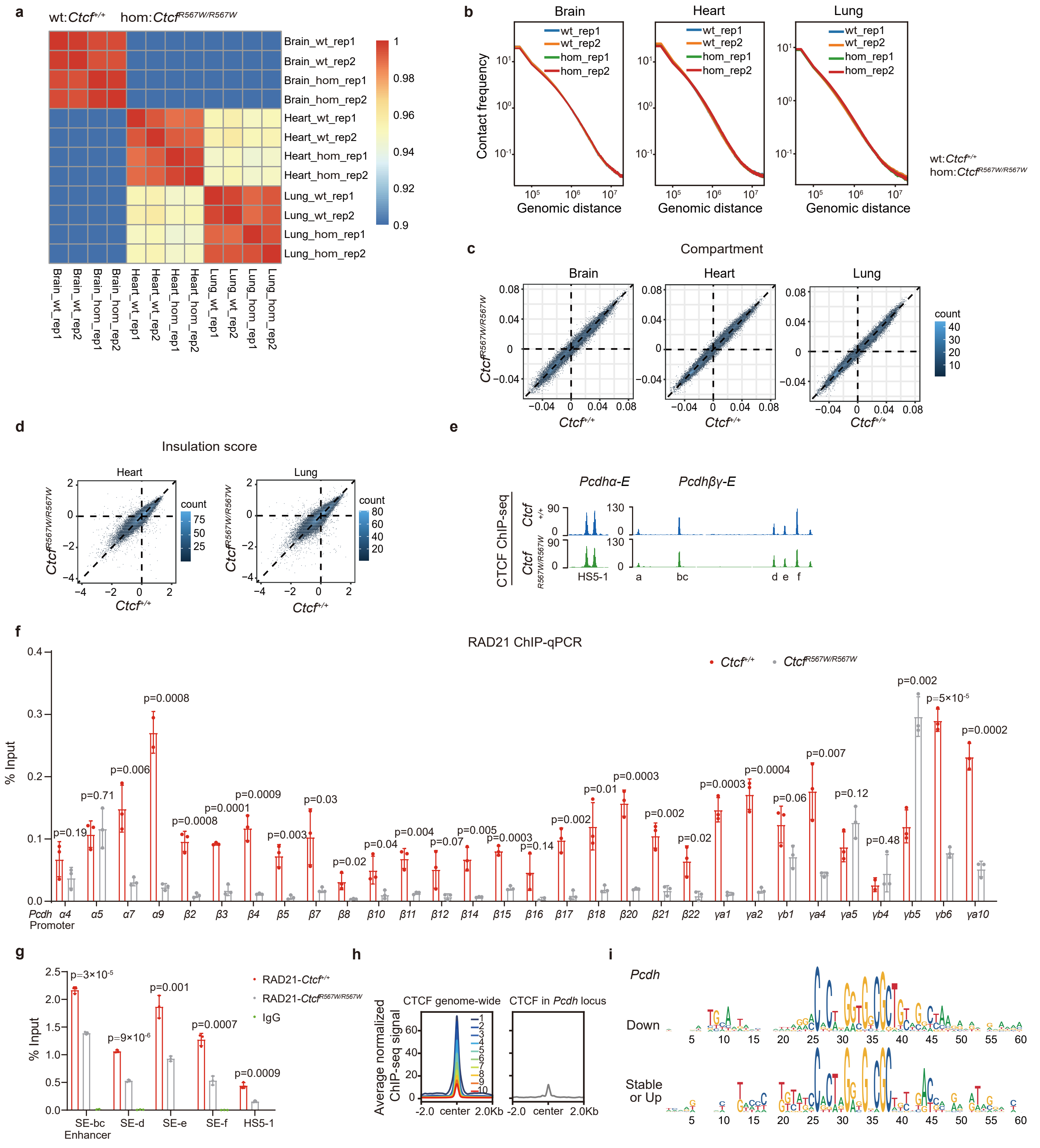

Supplementary Figure 8. Additional information on the regulatory mechanisms of CTCF^{R567W}. (**a**) Heatmap showing the correlation of BL-Hi-C data in different tissues. (**b**) Distance decay curves of BL-Hi-C data for brain, heart and lung tissues. (**c**) Comparison of compartment strength among brain, heart and lung tissues in *Ctcf*$^{+/+}$ and *Ctcf*$^{R567W/R567W}$ mice. The color in the plots represents the density of points. (**d**) Scatter plot showing the insulation scores in heart and lung tissues of *Ctcf*$^{+/+}$ and *Ctcf*$^{R567W/R567W}$ mice. The color in the plots represents the density of points. (**e**) Tracks showing CTCF binding in two enhancer regions of *cPcdh* in *Ctcf*$^{+/+}$ and *Ctcf*$^{R567W/R567W}$ brain tissues. (**f**, **g**) ChIP-qPCR analysis of RAD21 occupancy at the *cPcdh* locus (promoters of *cPcdh* genes in **f** and enhancers in **g**) in brain tissues of *Ctcf*$^{+/+}$ and *Ctcf*$^{R567W/R567W}$ mice. (**h**) Average CTCF binding strength for the indicated CTCF binding sites genome-wide or at the *cPcdh* locus. The genome-wide CTCF binding sites are splitted into 10 parts based on binding strength. The CTCF binding strength gradually weakens from 1 to 10. (**i**) Sequence logos showing the CTCF core motif sequences and ± 20 bp flanking sequences extracted from CTCF binding sites that exhibited altered or stable binding in response to the CTCF mutation. Quantitative data are presented as the mean ± SD. The *p*-values obtained by two-tailed unpaired *t*-test are indicated. Experiments in **f** and **g** were repeated independently twice with similar results. Source data are provided as a Source Data file.

Supplementary Fig. 9

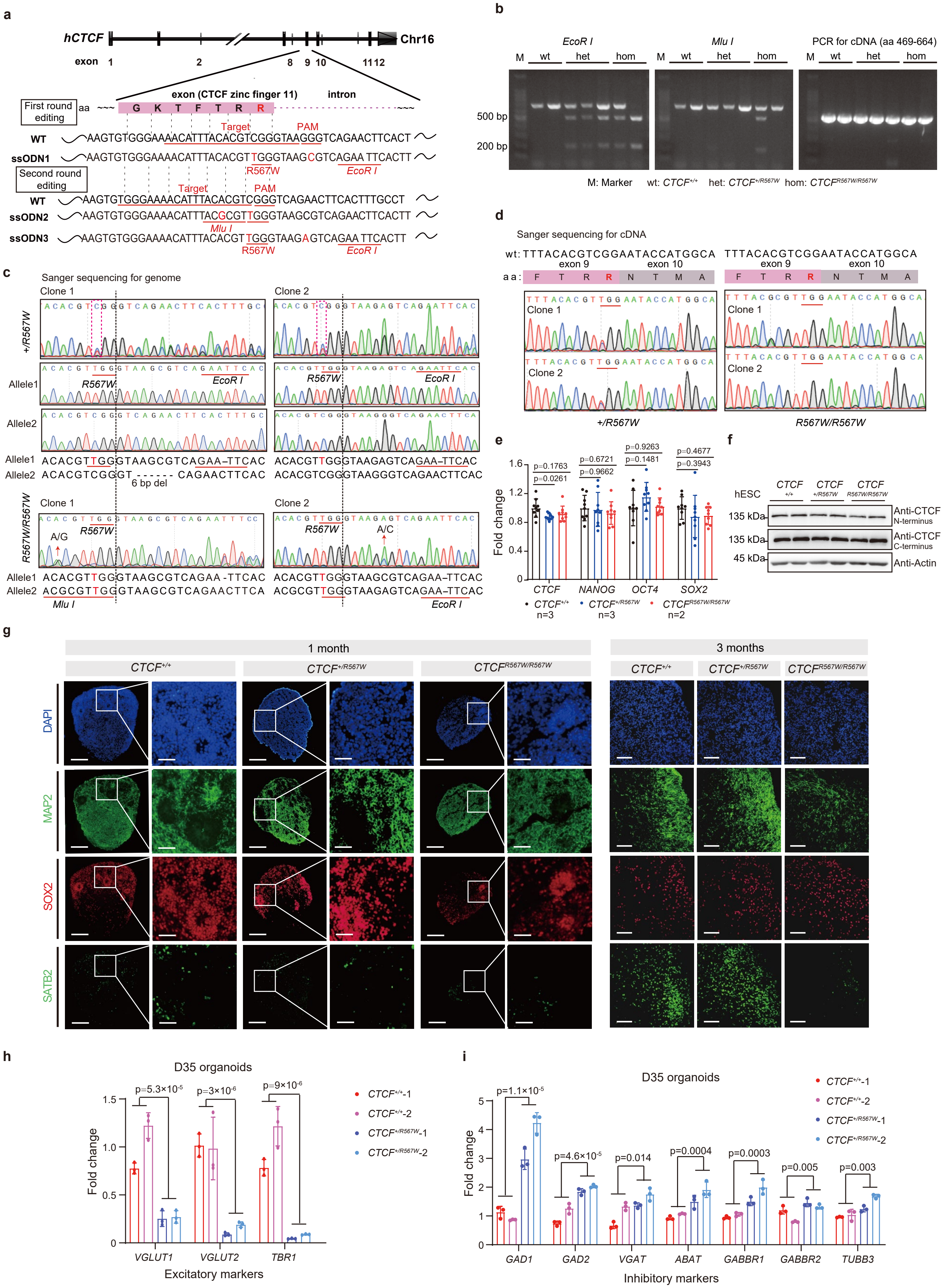

Supplementary Figure 9. Generation of CTCF^{R567W} hESC lines and the phenotype of cortical organoids. (**a**) Schematic diagram of the CRISPR/Cas9 mediated knock-in CTCF^{R567W} mutation in human H1 ESCs. The underline indicates the sgRNA targeting sequence (*CTCF* exon 9) and the mutation site R567W. The mutated base, PAM sequence NGG and enzyme site for identification are highlighted in red. (**b**) Gel images showing enzyme digestion of PCR products and cDNA amplification (aa 469-664) of the hESC mutation region. (**c**) Sanger sequencing results showing successful generation of *CTCF*$^{+/R567W}$ and *CTCF*$^{R567W/R567W}$ hESCs. The underline indicates the mutation base C > T and restriction enzyme digestion site. The sequences of each allele are listed below. The black dashed lines represent exon/intron boundaries. (**d**) Sanger sequencing results of cDNA (region: aa 469-664) from *CTCF*$^{+/R567W}$ and *CTCF*$^{R567W/R567W}$ hESCs. (**e**) RT-qPCR analysis of the expression of *CTCF* and pluripotency genes in *CTCF*$^{+/+}$, *CTCF*$^{+/R567W}$ and *CTCF*$^{R567W/R567W}$ hESCs (n = 3 clones for *CTCF*$^{+/+}$ and *CTCF*$^{+/R567W}$, n = 2 clones for *CTCF*$^{R567W/R567W}$). (**f**) Western blot analysis of CTCF protein levels in *CTCF*$^{+/+}$, *CTCF*$^{+/R567W}$ and *CTCF*$^{R567W/R567W}$ hESCs. (**g**) Immunofluorescence for the neuronal marker MAP2, dorsal forebrain neural progenitor marker SOX2, and CPN marker SATB2 in *CTCF*$^{+/+}$, *CTCF*$^{+/R567W}$ and *CTCF*$^{R567W/R567W}$ brain organoids at 1 month and 3 months after derivation. Scale bars: whole organoids (1 month), 200 μm; higher magnification (right column), 50 μm; others (3 months), 100 μm. CPN, callosal projection neurons. (**h**, **i**) RT-qPCR analysis of the expression of excitatory (**h**) and inhibitory (**i**) neuronal markers in *CTCF*$^{+/+}$ and *CTCF*$^{+/R567W}$ organoids on day 35 (n = 2 clones for each genotype). Quantitative data are presented as the mean ± SD. The *p*-values obtained by one-way ANOVA with Dunnett's multiple comparisons test (**e**) and two-tailed unpaired *t*-test (**h** and **i**) are indicated. For **e-i**, these experiments were repeated three times independently with similar results. Source data are provided as a Source Data file.

Supplementary Fig. 10

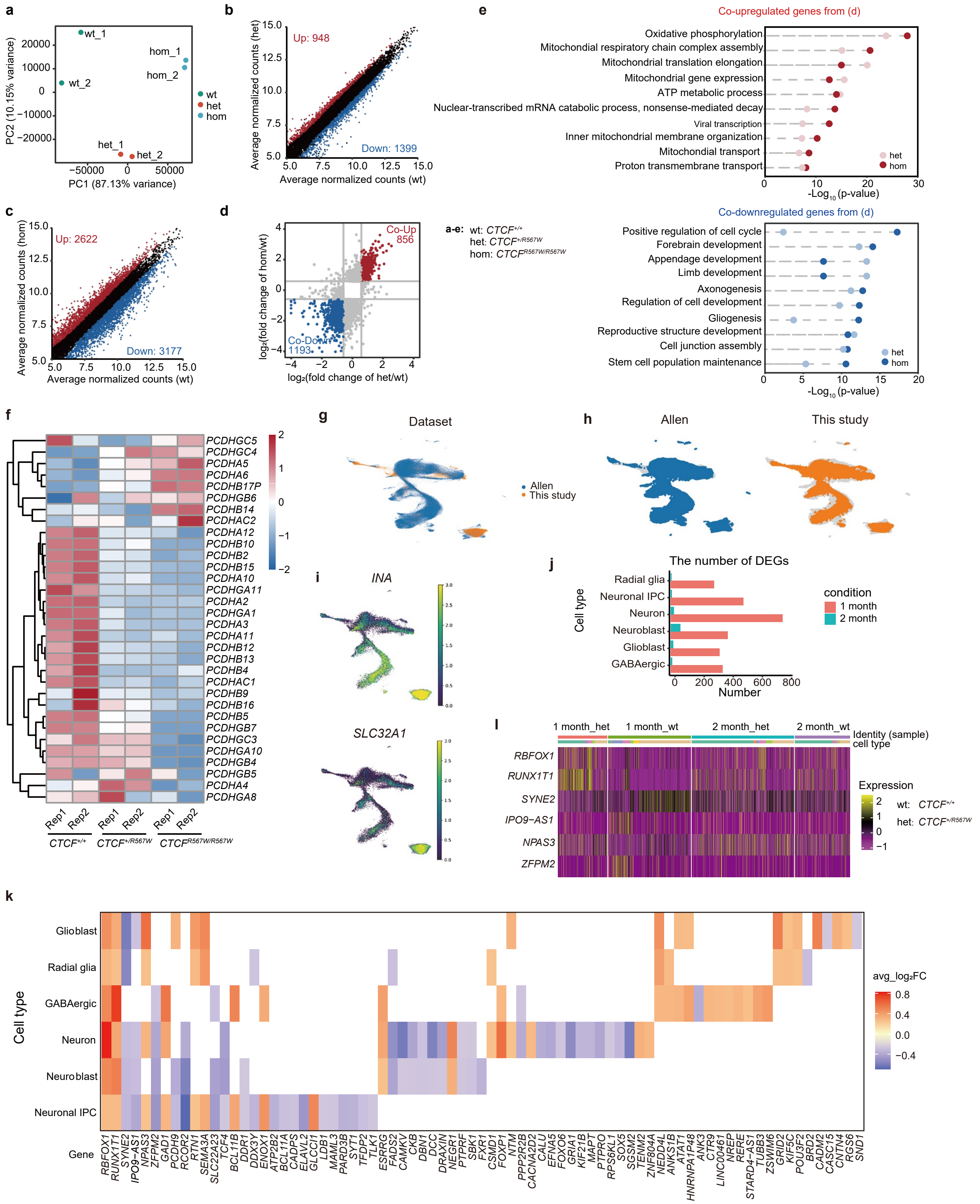

Supplementary Figure 10. Bulk RNA-seq and scRNA-seq data of cortical organoids. (**a**) PCA of RNA-seq data from *CTCF*$^{+/+}$, *CTCF*$^{+/R567W}$ and *CTCF*$^{R567W/R567W}$ cortical organoids on day 35. (**b**) Scatter plot showing the average normalized counts between *CTCF*$^{+/+}$ and *CTCF*$^{+/R567W}$ cortical organoids. Significantly upregulated genes are shown in red, and significantly downregulated genes are shown in blue. (**c**) Scatter plot showing the average normalized counts between *CTCF*$^{+/+}$ and *CTCF*$^{R567W/R567W}$ cortical organoids. Significantly upregulated genes are shown in red, and significantly downregulated genes are shown in blue. (**d**) Comparison of the $\log_2$(fold change) values between *CTCF*$^{+/+}$ and *CTCF*$^{+/R567W}$ cortical organoids with that between *CTCF*$^{+/+}$ and *CTCF*$^{R567W/R567W}$ cortical organoids. Co-upregulated genes are shown in red, and co-downregulated genes are shown in blue. Gray lines indicate $\log_2$(1.5). (**e**) Dot plots showing the top 10 common changed terms from GO results of DEGs between *CTCF*$^{+/+}$ and *CTCF*$^{+/R567W}$ cortical organoids and between *CTCF*$^{+/+}$ and *CTCF*$^{R567W/R567W}$ cortical organoids. (**f**) Heatmap showing the expression of *cPCDH* genes in three types of cortical organoids. (**g**, **h**) UMAP visualization of scRNA-seq data integrated from cortical organoids and fetal brain cortices, colored by dataset. (**i**) UMAP visualization of cortical organoid scRNA-seq data after integration, colored by the *INA* gene (a neuron marker) and *SLC32A1* gene (a GABAergic marker). (**j**) Histogram of the number of DEGs for each cell type in 1- and 2-month cortical organoid scRNA-seq data from the *CTCF*$^{+/+}$ and *CTCF*$^{+/R567W}$ groups. (**k**) Heatmap showing the DEGs from each cell type overlapping with the GWAS related to ASD, including autism and Asperger syndrome, in 1-month cortical organoid scRNA-seq data. (**l**) Heatmap showing the expression of DEGs associated with ASD from each cell type in 1- and 2-month cortical organoid scRNA-seq data from *CTCF*$^{+/+}$ and *CTCF*$^{+/R567W}$ groups.

Supplementary Table 1. List of sgRNA and ssODN sequences used for genome editing, related to Fig. 1 and Supplementary Fig. 9.

**sgRNA and ssODN for genome editing**	**Oligo sequences (5'- to -3')**
m-ssODN for *Ctcf* R567W mouse model	GATCCCAACTTTGTCCCTGCTGCCTTTGTCTGTTCCAAGTGTGGGAAA ACATTCACCCGCTGGGTAAGGCTCAGGCTCGCTGTTATGGCTCTTAAT AGCACACACTTGATCTTCAGTTACAGAATGGAGTTGTGGCCACTGGAG
sgRNA for *Ctcf* R567W mouse model	AAACATTCACCCGCCGGGTAAGG
h-ssODN-1 for *CTCF* R567W hESCs	ACTTCGTCCCTGCGGCTTTTGTCTGTTCTAAGTGTGGGAAAACATTTAC ACGTTGGGTAAGCGTCAGAATTCACTTTGCCTGTTATGATACTGAATAT TGGATTTTTGGTCTTCCT
h-ssODN-2 for *CTCF* R567W hESCs	ACTTCGTCCCTGCGGCTTTTGTCTGTTCTAAGTGTGGGAAAACATTTAC GCGTTGGGTAAGCGTCAGAACTTCACTTTGCCTGTTATGATACTGAAT ATTGGATTTTTGGTCTTCCT
h-ssODN-3 for CTCF R567W hESCs	ACTTCGTCCCTGCGGCTTTTGTCTGTTCTAAGTGTGGGAAAACATTTAC ACGTTGGGTAAGAGTCAGAATTCACTTTGCCTGTTATGATACTGAATAT TGGATTTTTGGTCTTCCT
sgRNA for *CTCF* R567W hESCs-1	AACATTTACACGTCGGGTAA
sgRNA for *CTCF* R567W hESCs-2	TGGGAAAACATTTACACGTC

Supplementary Table 2. List of primers used for genotyping, Hi-C and QHR-4C experiments, related to Fig. 1, Fig. 5, and Supplementary Fig. 9.

**Genotyping and sequencing primers**	
**Primer name**	**Primer sequences (5'- to -3')**
U6 Forward Primer	GACTATCATATGCTTACCGT
Mouse genotyping Primer-F	GATGATAGCCCTGTGACTCTCGGAG
Mouse genotyping Primer-R	AGATTTCAATCCACGCAAGGAAATG
hESCs-genotyping Primer-1-F	AACAAGCCGTGTGGAGTCTA
hESCs-genotyping Primer-1-R	GATACTGTGTTCCCCATCAGTGG
hESCs-genotyping Primer-2-F	CCCTATGCCGTTTCAGGAGA
hESCs-genotyping Primer-2-R	GGTGCAGGTTTCAATCCACC
hESCs-genotyping (cDNA aa 469-644) -F	TGCCGTTACTGTGATGCTGT
hESCs-genotyping (cDNA aa 469-644) -R	TGTTTGGGCTGGTTGGTTCT
Mouse-genotyping sequencing	GATGATAGCCCTGTGACTCTCGGAG
hESCs-genotyping sequencing-1	AACAAGCCGTGTGGAGTCTA
hESCs-genotyping sequencing-2	CCCTATGCCGTTTCAGGAGA
**Primers used for Hi-C and QHR-4C**	
**Primer name**	**Primer sequences (5'- to -3')**
BL-linker-F	P-CGCGATATC/iBIOdT/TATCTGACT
BL-linker-R	P-GTCAGATAAGATATCGCGT
Adapter-U	GACGTGTGCTCTTCCGATCTGNNNNNNNN-NH2
Adapter-L	P-CAGATCGGAAGAGCACACGTC-NH2
biotin-m-se-bc	5’ BIOTIN-AACTATGCTCAGGGCCTCTGG
biotin-m-se-f	5’ BIOTIN-CTCCATGTGCCATCTGGTGG
qhr-m-se-bc-F1	AATGATACGGCGACCACCGAGATCTACACTCTTTCCCTACACGAC GCTCTTCCGATCTGAGGCTTATGGGCCTTACAGATC
qhr-m-se-f-F1	AATGATACGGCGACCACCGAGATCTACACTCTTTCCCTACACGAC GCTCTTCCGATCTGGAGCACTGTTGCTTAGAGATTCTC
qhr-P7-R1	CAAGCAGAAGACGGCATACGAGATACATCGGTGACTGGAGTTCA GACGTGTGCTCTTCCGATCT
qhr-P7-R2	CAAGCAGAAGACGGCATACGAGATTGGTCAGTGACTGGAGTTCA GACGTGTGCTCTTCCGATCT
qhr-P7-R3	CAAGCAGAAGACGGCATACGAGATCACTGTGTGACTGGAGTTCA GACGTGTGCTCTTCCGATCT
qhr-P7-R4	CAAGCAGAAGACGGCATACGAGATATTGGCGTGACTGGAGTTCA GACGTGTGCTCTTCCGATCT
qhr-P7-R5	CAAGCAGAAGACGGCATACGAGATGATCTGGTGACTGGAGTTCA GACGTGTGCTCTTCCGATCT
qhr-P7-R6	CAAGCAGAAGACGGCATACGAGATTACAAGGTGACTGGAGTTCA GACGTGTGCTCTTCCGATCT

Supplementary Table 3. List of primers used for RT-qPCR experiments, related to Supplementary Fig. 1 and Supplementary Fig. 9.

Primer name	Forward primer (5'- to -3')	Reverse primer (5'- to -3')
mGapdh	AGGTCGGTGTGAACGGATTTG	TGTAGACCATGTAGTTGAGGTCA
mCtcf	GATCCTACCCTTCTCCAGATGAA	GTACCGTCACAGGAACAGGT
mGH	GCTACAGACTCTCGGACCTC	CGGAGCACAGCATTAGAAAACAG
hCTCF	ATCACGACCCCAACTTCGTC	GGCTCTGGCTCAGGTTCAAT
hOCT4	CCTCACTTCACTGCACTGTA	CAGGTTTTCTTTCCCTAGCT
hSOX2	CCCAGCAGACTTCACATGT	CCTCCCATTTCCCTCGTTTT
hNANOG	TGAACCTCAGCTACAAACAG	TGGTGGTAGGAAGAGTAAAG
hTBR1	GCAGCAGCTACCCACATTCA	AGGTTGTCAGTGGTCGAGATA
hVGLUT1	CAGAGTTTTCGGCTTTGCTATTG	GCGACTCCGTTCTAAGGGTG
hVGLUT2	GGGAGACAATCGAGCTGACG	TGCAGCGGATACCGAAGGA
hVGAT	ACGTCCGTGTCCAACAAGTC	AAAGTCGAGGTCGTCGCAATG
hGAD1	GCGGACCCCAATACCACTAAC	CACAAGGCGACTCTTCTCTTC
hGAD2	TTTTGGTCTTTCGGGTCGGAA	TTCTCGGCGTCTCCGTAGAG
hABAT	AAGAGAGCCGAGGCAATTACC	GCTCGCATTTTGAGGCTGTTG
hGABBR1	GAGGACGTGAATAGCCGCAG	CTGGATCACACTTGCTGTCGT
hGABBR2	CCGCAACGAGTCACTCCTG	CAAGTGGTTAGGCCCGTATTTTA
hTUBB3	GGCCAAGGGTCACTACACG	GCAGTCGCAGTTTTCACACTC
hGAPDH	GGTGGTCTCCTCTGACTTCAAC	GTTGCTGTAGCCAAATTCGTTGT

Supplementary Table 4. List of primers and probes used for EMSA/GST-pull down assays, related to Supplementary Fig. 6.

Primers used for CTCF ZF purification	
**Primer name**	**Primer sequences (5'- to -3')**
pGEX-aa260-ZF-F	CGTGGATCCCCAGGAAAAGGTGTAAAGAAA
pGEX-aa583-ZF-R	TGCGGCCGCTCGAGTTACGCCATCTGGACC
Mut (R567W)-ZF-F	TTCACCCGCTGGAACACAATGGCAAGACAT
Mut (R567W)-ZF-R	ATGTCTTGCCATTGTGTTCCAGCGGGTGAA
Mut (R566C R567W)-ZF-F	GAAAACATTCACCTGCTGGAACACAATGGC
Mut (R566C R567W)-ZF-R	GCCATTGTGTTCCAGCAGGTGAATGTTTTC
**Probes of CTCF motif**	**Oligo sequences (5'- to -3')**
M1-F (cy5 or biotin labeled) / (C motif)	CTTTTTGGTGCCCTCTGCTGGCCAGTTTAG
M1-R / (C motif)	CTAAACTGGCCAGCAGAGGGCACCAAAAAG
M2-F (cy5 or biotin labeled) / (U motif)	CTTTTGGAACTGCAGTTTAG
M2-R / (U motif)	CTAAACTGCAGTTCCAAAAG
M1+M2-F (cy5 or biotin labeled) / (U+C motif)	CTTTTTGGTGCCCTCTGCTGGCCACTGGAGGAACTGCAG TTTAG
M1+M2-R / (U+C motif)	CTAAACTGCAGTTCCTCCAGTGGCCAGCAGAGGGCACCA AAAAG
M1+M2 (mut)-F (cy5 or biotin labeled) / (mutant U+C motif)	CTTTTTGGTGCCCTCTGCTGGCCACTGGAGGACCGAAAG TTTAG
M1+M2 (mut)-R / (mutant U+C motif)	CTAAACTTTCGGTCCTCCAGTGGCCAGCAGAGGGCACCA AAAAG

Supplementary Table 5. List of primers used for ChIP-qPCR experiments, related to Supplementary Fig. 8.

Primer name	Forward primer (5'- to -3')	Reverse primer (5'- to -3')
ChIPQ-SE-bc	TTTGCTTGACCCAGGCACTT	CCAGGGCTTTACCCTTCGTA
ChIPQ-SE-d	AAATGGAGTTAGCTGGGGGC	AAAAACCCGGAGTCCCTGTG
ChIPQ-SE-e	TCATCTGATCGGCTCCTCCA	GTTTTCCTTTTGGCCACGGT
ChIPQ-SE-f	AAATAACCCTCCTGGTCGGC	ACGTTAGAGAGCGACCTCCT
ChIPQ-HS5-1	ATGACAGCTTCCGGTAGGGC	CCAGCACTTTCCTCATCGAAC
ChIPQ-Pcdha7	ATGATGTCGCTCTTAGCCACTA	GCCATTGTGGAGCAACCGAA
ChIPQ-Pcdha9	TTAGCCACTGGATGTCGCTG	CCGAGATCTCCGAACGTAGC
ChIPQ-Pcdhb2	TAAAAGGAACCGGCGATCAG	GAAGGTTTCCTCTCCCCAACG
ChIPQ-Pcdhb3	ATACTGAGAACGAACGCATGG	AAGCAGTGGTTTTACATCATCGG
ChIPQ-Pcdhb4	AGTGAATAAGGACGCTTGGTG	TGATTCGGTAATGGCTTCCAGG
ChIPQ-Pcdhb5	TGCAGGCTAAGGTTTGTGAAA	AACCCATTCAAGGTCGGGTATT
ChIPQ-Pcdhb7	TTTATCAAAGTGGACGCAGGGT	TTGCAACAAGCGATTCCGGG
ChIPQ-Pcdhb8	GTAGGGCTGGAACTGAGAGC	CTGATTCCCAGGAATGGCGT
ChIPQ-Pcdhb10	GCGCTGTCGACTAAGGGAG	TCCCCTAGATCACGCTCAAA
ChIPQ-Pcdhb11	TGCAGGCTTAAGAGTGTCAAGAA	GCCATCGTTCTGTCTCGACTT
ChIPQ-Pcdhb12	ACACTTCAGGCCACTTGTGTAA	AATGATCACACAAACCCTACCCA
ChIPQ-Pcdhb14	AAGTGGGCCACACATTTTAGC	TCTGAAAGGATCGATCGGCAG
ChIPQ-Pcdhb15	TAAACACTGGTGAGGAGTGC	CCTTGCGTTTTGTGAGTGCTA
ChIPQ-Pcdhb16	GCCCACTGCCAGATAAAGGT	TTCAGTCCTTTTCCGAGGGC
ChIPQ-Pcdhb17	TGTAAGGCTCCGTGAGACCG	TGGCCGAAAAAGACACACCC
ChIPQ-Pcdhb18	AGGGAGATTAAAGTGAGTCTGCG	CTTTCCCCCTCATCGGTCAGA
ChIPQ-Pcdhb20	CACATGGTGGCGCTATAGGATA	TGGAATCGTCTGTTCTCTGGTC
ChIPQ-Pcdhb21	AATGTTTCTCAACAGCAGCTCG	GAGTGCTGTTTTATCCAGTGCC
ChIPQ-Pcdhb22	TGAAGAGCATAGTCAACGCA	TCCCAAGTGTAGATTAGCAGCA
ChIPQ-Pcdhga1	GCATCTTCAAGGAGTGCAGTAAC	CTAAGAGTCACGCGTTTCCCC
ChIPQ-Pcdhga2	GCTGTTGACCACCTAAGGAGT	GAAGCTGCGGACAAACTACC
ChIPQ-Pcdhga4	ACCACTCAGGGGTGAAAATCAG	ATCATCACCAGTCCTTTGTGCT
ChIPQ-Pcdhga10	TCTTCCGACTCTGAGCGCC	TGGACCCTGAGAATCCCAACT
ChIPQ-Pcdhga5	CTAAGCGTCGCTGTTCACCA	GGAGAGCGGGACTAAGGATG
ChIPQ-Pcdhgb1	GCTGCACGATACGCAGTTTT	CGGATGTTCTCATGGGTGCT
ChIPQ-Pcdhgb4	TTGCTAGGCTGCAGTTTCCT	TGCTCAGAAAGAAGCGGTGT
ChIPQ-Pcdhgb5	TTGGGATGGGGACCAAACAG	GGAGCCGTAGAAAACGCTCT
ChIPQ-Pcdhgb6	CTGCAGTTCCACTCAGAGCC	GAGTTCCCCTTGGTGTGTGT
ChIPQ-Pcdha4	GTTTCCACTAGAGGGCGCTT	TACCCGTTCGTCGTTGGATG
ChIPQ-Pcdha5	ACCCTTTTATCCACATGGTGTCG	GTGAGCTTCTGTAGCAGATCCAA

Supplementary Table 6: List of antibodies used in this study.

Immunofluorescence				
**Primary Antibody**	**Host**	**Manufacturer**	**Catalog Number**	**Dilution**
SATB2	Mouse	Abcam	Cat# AB51502	1:200
MAP2	Mouse	Sigma-Aldrich	Cat# M4403	1:100
SOX2	Rabbit	Cell Signaling Technology	Cat# 23064	1:200
βIII-tublin (TUJ1)	Rabbit	Abcam	Cat# AB18207	1:200
**Secondary Antibody**	**Host**	**Manufacturer**	**Catalog Number**	**Dilution**
Alexa Fluor™ 594 goat anti rabbit IgG (H+L)	Goat	Thermo Fisher Scientific	Cat# A11012	1:300
Alexa Fluor™ 488 goat anti mouse IgG (H+L)	Goat	Thermo Fisher Scientific	Cat# A11001	1:300
Alexa Fluor™ 488 goat anti rabbit IgG (H+L)	Goat	Thermo Fisher Scientific	Cat# A11008	1:300
**Immunohistochemistry**				
**Primary Antibody**	**Host**	**Manufacturer**	**Catalog Number**	**Dilution**
CTCF	Rabbit	Active motif	Cat# 61311	1:500
**Secondary Antibody**	**Host**	**Manufacturer**	**Catalog Number**	**Dilution**
Boost IHC Detection Reagent (HRP, Rabbit)	Goat	Cell Signaling Technology	Cat# 8114	——
**Western blot**				
**Primary Antibody**	**Host**	**Manufacturer**	**Catalog Number**	**Dilution**
CTCF (C-terminus)	Rabbit	Millipore	Cat# 07-729	1:1000
CTCF (N-terminus)	Mouse	Abcam	Cat# ab37477	1:1000
β-Actin	Mouse	Sigma-Aldrich	Cat# A2228	1:5000
**Secondary Antibody**	**Host**	**Manufacturer**	**Catalog Number**	**Dilution**
HRP Anti-Mouse IgG H&L	Goat	KangChen	Cat# KC-MM-035	1:5000
HRP Anti-Rabbit IgG H&L	Goat	KangChen	Cat# KC-RB-035	1:5000
**ChIP**				
**Primary Antibody**	**Host**	**Manufacturer**	**Catalog Number**	**Dilution**
CTCF	Rabbit	Active motif	Cat# 61311	1:200
RAD21	Rabbit	Abcam	Cat# ab217678	1:200
IgG	Rabbit	Abcam	Cat# ab37415	1:1000

Uncropped gels and blots

Related to Supplementary Fig. 1f

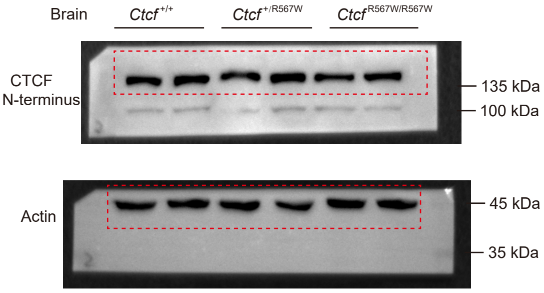

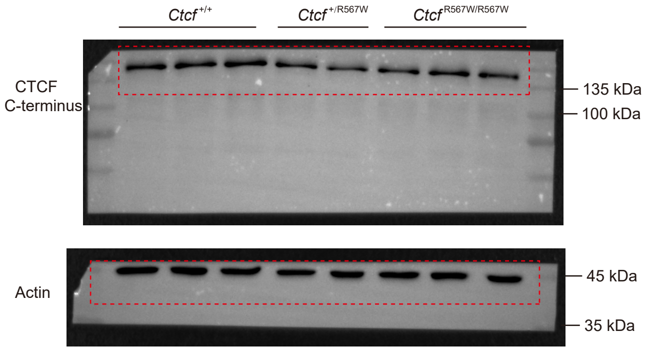

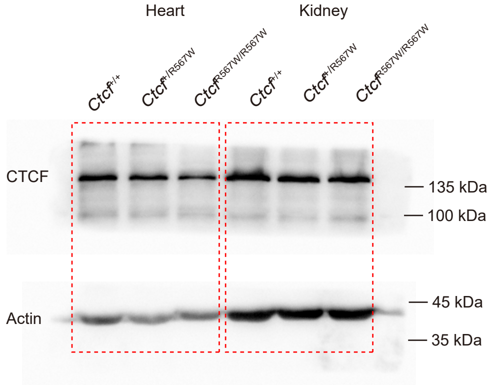

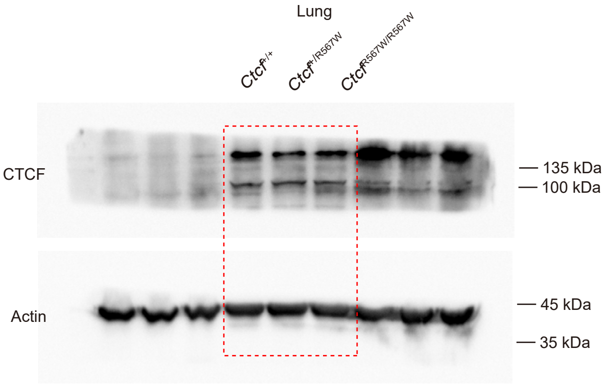

Related to Supplementary Fig. 6a

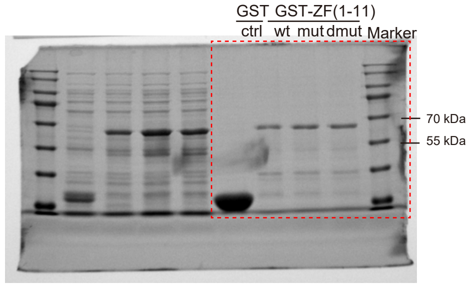

Related to Supplementary Fig. 6d

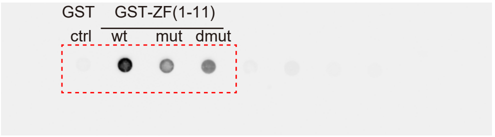

Related to Supplementary Fig. 6e

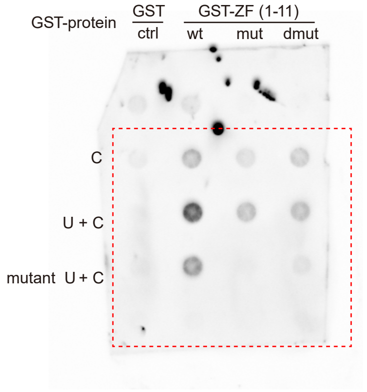

Related to Supplementary Fig. 9b

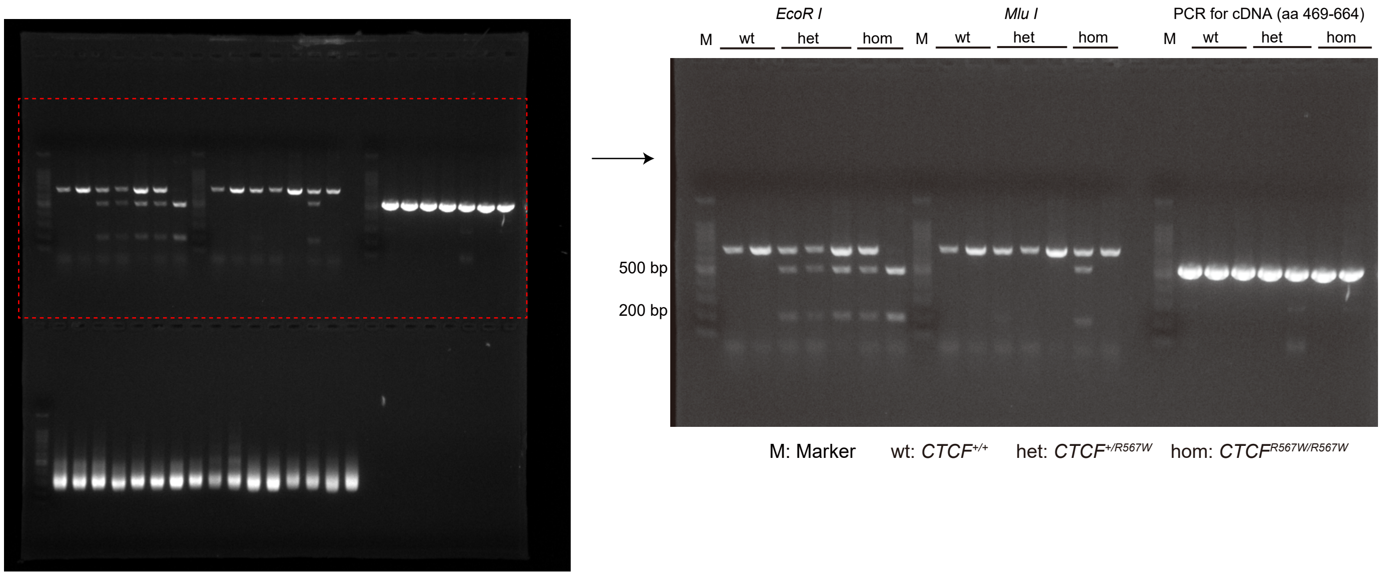

Related to Supplementary Fig. 9f

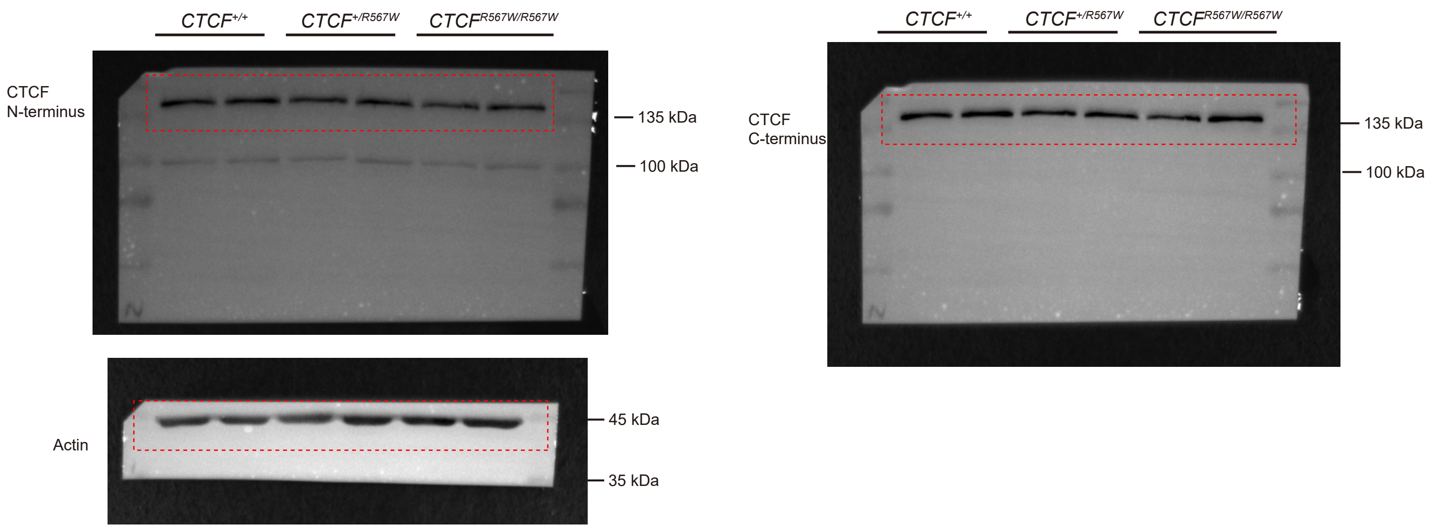

Supplement: Supplementary file 1 — Supplementary information [file 41467_2024_49684_MOESM1_ESM.pdf]
