# Supplementary material for: CTCF mutation at R567 causes developmental disorders via 3D genome rearrangement and abnormal neurodevelopment
Source: Nat Commun. 2024 Jul 1;15:5524. doi: 10.1038/s41467-024-49684-1 (PMC11217373; doi:10.1038/s41467-024-49684-1)
Supplement: Supplementary file 3 — Description of Additional Supplementary Files [file 41467_2024_49684_MOESM3_ESM.pdf]

Description of Additional Supplementary Files

Supplementary Data 1.
DEGs from brain tissues and neurons.

Supplementary Data 2.
DEGs from cortical organoids RNA-seq data.

Supplementary Data 3.
DEGs from cortex snRNA-seq data.

Supplementary Data 4.
DEGs from heart snRNA-seq data.

Supplementary Data 5.
DEGs from lung snRNA-seq data.

Supplementary Data 6.
CTCF peaks in brain, heart, and lung tissues.

Supplementary Data 7.
Quality control statistics of BL-Hi-C data and TAD boundaries.

Supplementary Data 8.
DEGs from organoid scRNA-seq data.
