# Supplementary material for: CTCF mutation at R567 causes developmental disorders via 3D genome rearrangement and abnormal neurodevelopment
Source: Nat Commun. 2024 Jul 1;15:5524. doi: 10.1038/s41467-024-49684-1 (PMC11217373; doi:10.1038/s41467-024-49684-1)
Supplement: Supplementary file 12 — Reporting Summary [file 41467_2024_49684_MOESM12_ESM.pdf]

nature portfolio

Corresponding author(s): Hongjie Yao

Last updated by author(s): May 13, 2024

Reporting Summary

Nature Portfolio wishes to improve the reproducibility of the work that we publish. This form provides structure for consistency and transparency in reporting. For further information on Nature Portfolio policies, see our Editorial Policies and the Editorial Policy Checklist.

Statistics

For all statistical analyses, confirm that the following items are present in the figure legend, table legend, main text, or Methods section.

n/a	Confirmed	
☐	☒	The exact sample size (*n*) for each experimental group/condition, given as a discrete number and unit of measurement
☐	☒	A statement on whether measurements were taken from distinct samples or whether the same sample was measured repeatedly
☐	☒	The statistical test(s) used AND whether they are one- or two-sided<br>*Only common tests should be described solely by name; describe more complex techniques in the Methods section.*
☒	☐	A description of all covariates tested
☐	☒	A description of any assumptions or corrections, such as tests of normality and adjustment for multiple comparisons
☐	☒	A full description of the statistical parameters including central tendency (e.g. means) or other basic estimates (e.g. regression coefficient) AND variation (e.g. standard deviation) or associated estimates of uncertainty (e.g. confidence intervals)
☐	☒	For null hypothesis testing, the test statistic (e.g. *F*, *t*, *r*) with confidence intervals, effect sizes, degrees of freedom and *P* value noted<br>*Give P values as exact values whenever suitable.*
☒	☐	For Bayesian analysis, information on the choice of priors and Markov chain Monte Carlo settings
☒	☐	For hierarchical and complex designs, identification of the appropriate level for tests and full reporting of outcomes
☒	☐	Estimates of effect sizes (e.g. Cohen's *d*, Pearson's *r*), indicating how they were calculated

Our web collection on statistics for biologists contains articles on many of the points above.

Software and code

Policy information about availability of computer code

Data collection

Histological images were acquired with a Pannoramic MIDI slice scanner (3D HISTECH). Immunofluorescence images were acquired with LSM 800/710 NLO laser scanning confocal microscope (Carl Zeiss). Quantitative PCR was performed in a CFX96 Real-Time System (Bio-Rad). Multi-electrode array (MEA) measurements were recorded by Maestro Pro instrument and AxIS Navigator software. Mouse behavioral tests were recorded by video tracking software EthoVision XT 13 (Noldus). Electrophoretic mobility shift assay (EMSA) gels were scanned in a gel imaging system (Azure Biosystems). RNA-seq, snRNA-seq, ChIP-seq, BL-Hi-C, QHR-4C libraries were sequenced on Illumina NovaSeq. scRNA-seq libraries of cortical organoids were sequenced on the DNBSEQ-T7RS platform.

Data analysis

The recorded images were quantified with ImageJ software (v8.0.2). Statistical analyses were performed using GraphPad Prism9 software (v9.0.0). Adobe Illustrator (23.0.1) was used to prepare figures. For sequencing data analysis of RNA-seq, snRNA-seq, ChIP-seq, BL-Hi-C, QHR-4C, and scRNA-seq, our data analysis was described in the manuscript.

RNA-seq and data analysis: paired-end reads were handled with Trim Galore (version 0.6.5) and then mapped to the mouse (mm10) or human (hg38) genome using STAR aligner (version 2.7.0). Transcript abundance was quantified by RSEM (version 1.2.22). DEGs were determined using DESeq2 (version 1.32.0) with q value < 0.05 and fold-change > 1.5. Gene Ontology (GO) analysis was performed using clusterProfiler (version 4.0.0).

SnRNA-seq and data analysis for mouse brain cortex: the BD Rhapsody whole transcriptome analysis (WTA) pipeline was utilized to process FASTQ files, converting raw sequencing reads into gene expression matrix with unique molecular identifier (UMI) counts for each gene in each cell. The Seurat (version 4.0.5) was utilized for further analysis of the read count matrix for each gene/sample. Quality control measures were applied to filter individual cells, retaining only those expressing genes detected in at least 3 cells and cells expressing a minimum of 200 genes. For cell annotation, the ScType package was used. To identify genes showing differential expression between CTCF mutated sample and wild

type sample, the Seurat package's FindMarkers function was employed. Differential expression analysis utilized the Wilcox method, which applies the Wilcoxon rank-sum test to compare gene expression levels between groups of cells. Benjamini-Hochberg method was used for multiple testing correction of p-values to ensure the rigour of the statistical results. A corrected p-value less than 0.05 with log2 fold change above 0.5 was considered statistically significant. All significant DEGs in each cell type were selected for GO enrichment analysis using clusterProfiler (v3.18.1).

SnRNA-seq and data analysis for mouse heart and lung tissues: paired-end reads were aligned to the mouse reference genome (mm10) using Cell Ranger (version 6.1.1). Raw count matrices were used to remove ambient RNA with SoupX (version 1.6.1) and then analyzed using Seurat (version 4.1.1). Poor-quality cells (> 50% mitochondrial genes, < 500 genes and < 500 UMI per cell for heart samples; > 5% mitochondrial genes, < 500 genes and < 1,000 UMI per cell for lung samples) were filtered out. Potential doublets were removed with DoubletFinder (version 2.0.3). The data were then normalized and integrated using the SCTransform integration workflow by regressing out the cell cycle effect and percentages of mitochondria-expressed genes per cell. PCA was performed (npcs = 50), and dimensionality reduction (uniform manifold approximation and projection, UMAP) and clustering were then carried out (dimensions=30; cluster resolution=0.2 for heart samples, 0.4 for lung samples). Cell markers for each cell cluster were determined with the FindAllMarkers function. For heart samples, cell types were annotated based on a reference paper. For lung samples, cell types were manually verified based on canonical markers. DEGs were identified via FindMarkers with a min. pct = 0.25 and an adjusted p-value < 0.05. GO networks for DEGs were constructed using BinGo (version 3.0.5)66 and then visualized in Cytoscape (version 3.9.1).

ChIP-seq data analysis: Raw reads were trimmed to remove adaptors using Trim Galore and then mapped to the mouse (mm10) and human (hg38) mixed genomes using Bowtie2 (version 2.2.5) with the following parameters: --very-sensitive --end-to-end --no-unal --no-mixed --no-discordant. Only uniquely mapped reads with MAPQ > 30 were retained. Reads mapped to the mouse or human genome were separated and then subjected to peak calling using macs2 (version 2.2.7.1) with the default options. To quantitatively compare the CTCF binding strength between wild-type and CTCF-mutated samples, CTCF ChIP signals were normalized according to the method in a previously published paper with minor changes. Normalization was performed based on the assumption that the average CTCF binding strength from HEK293T cells mixed in different samples should be the same. The peak summit value of the averaged CTCF binding strength from HEK293T cells for each sample was extracted to calculate the scale factor. The scale factor for wild-type samples was considered to be 1. For corresponding CTCF-mutated samples, the scale factor was calculated by summit (wild-type)/summit (mutant). The scale factors for brain, heart and lung samples are shown in Supplementary Fig. 7a. Normalized bigwig files were generated with the bamCoverage tool in deepTools2 (version 3.5.1) using RPGC normalization and scale factors.

CTCF upstream motif analysis: The CTCF core motif was first identified for each CTCF site with FIMO (version 5.5.3) using the parameter "--thresh 1e-4" with the CTCF position frequency matrix obtained from the HOCOMOCO database, and then the upstream 20 bp sequence for each CTCF site was extracted. The upstream sequences were clustered using hamming distance. Heatmaps were generated with ggmsa (version 1.3.4), and sequence logos were generated with ggseqlogo (version 0.1).

BL-Hi-C data analysis: The adaptors were trimmed from raw paired-end reads with Trim Galore, and the linker sequences were removed with the trimlinker tool from the ChIA-PET2 package (version 0.9.3). The trimmed sequences were then mapped to the mouse (mm10) genome and processed using HiC-Pro (version 2.11.1). The reproducibility of the Hi-C replicates was assessed with HiCRep (version 0.2.6). The valid pair files generated by HiC-Pro were transformed into hic files with KR normalization using juicer tools (version 1.122.01). A/B compartments were analyzed by juicer tools with 100 kb resolution. Insulation scores (ISs) were calculated with 50 kb resolution using FAN-C (version 0.9.20). ISs were calculated with a window size of 400 kb for brain samples and a window size of 300 kb for heart and lung samples. TAD boundaries were determined by the "fanc boundaries" function with the parameter -s 0.7. The genomic regions that had no mapped reads and the lowest 5% covered reads were combined as low-coverage regions, which were excluded from the insulation score and TAD boundary analysis. The region between two nearby TAD boundaries was defined as the TAD.

QHR-4C data analysis: Adaptor sequences in raw paired-end reads were removed with Trim Galore. The primer sequence at the 5' end of read 1 was then trimmed with cutadapt (version 3.4). Reads that did not contain primer sequences were discarded. Reads were mapped to the mm10 genome using Bowtie2 with the following parameters: --very-sensitive --end-to-end --no-unal -X 2000. Bam files were imported into the r3Cseq package (version 1.38.0). Normalized bedgraph files were thus generated and then transformed into bigwig files using the bedGraphToBigWig tool.

Organoid scRNA-seq data analysis: Cell Ranger (version 6.1.1) with the human reference genome (hg38) was used to generate the output count matrix. Cells with higher than 20% mitochondrial content, fewer than 300 features, and features expressed in fewer than three cells were excluded from the analysis. We used Scrublet (version 0.2.3) and the gene expression patterns (total counts > 40000) to determine the doublets. The doublet score was calculated independently for each sample with the default settings and with the expected doublet score set at a rate at 10%. The cortex region data were extracted from the Allen Brain's human fetal developmental brain scRNA data, which were used as reference data for cell type annotation. SCANPY (version 1.9.1) and Seurat (version 4.3.0) were used for major data analysis and visualization. The following steps were performed in order: data normalization, log-transformation, highly variable gene selection, and PCA. The expression levels were calculated as counts per 10,000 (NC); briefly, the total mapped read counts of a gene within one cell were scaled by the number of total mapped reads of that cell and times multiplied by 10,000. The log2 (NC + 1)-transformed values were used for further analysis unless indicated otherwise. The highly variable genes were selected based on the raw count data with flavor as "Seurat_v3". The top 1000 were selected. PCA was run with the selected highly variable genes. Data integration was performed on the top highly variable genes via Harmony (version 0.0.6) with the default parameters. UMAP was performed on the Harmony-corrected PCA embedding. The neighborhood graphs were calculated using scanpy.pp.neighbors, with 15 local neighborhoods and 50 Harmony-corrected PCAs using the mKNN graph. The connectivities were computed using the UMAP method with Euclidean distance. Then, UMAP embedding was performed using scanpy.tl.umap with a minimal effective distance of 0.5, a spread of 1.0, an initial learning rate of 1.0, a negative sample weighting of 1.0 and a negative edge sample rate of 5. Louvain clustering was performed using sc.tl.louvain on the neighborhood calculated in the previous steps, with a resolution of 1, with the vtraag package and with no weights from the mKNN graph. For each cluster, a logistic regression model was trained on the reference dataset and used to annotate organoid cell types. After cell type annotation, the proportions of various cells in each sample were calculated. Milopy (version 0.1.1) was used to calculate and visualize the changes in cell proportions between wild-type and CTCF-mutated cells in one-month samples. DEGs for each cell type population between wild-type and CTCF-mutated samples (see Supplementary Data 8) were selected by Seurat via FindMarkers with min. pct = 0.25 and an adjusted p-value < 0.05. GO enrichment analysis was performed on the sets of differentially expressed genes for each cell type using the enrichGO function in the clusterProfiler (version 3.18.1) package.

For manuscripts utilizing custom algorithms or software that are central to the research but not yet described in published literature, software must be made available to editors and reviewers. We strongly encourage code deposition in a community repository (e.g. GitHub). See the Nature Portfolio guidelines for submitting code & software for further information.

Data

Policy information about availability of data

All manuscripts must include a data availability statement. This statement should provide the following information, where applicable:

- Accession codes, unique identifiers, or web links for publicly available datasets
- A description of any restrictions on data availability
- For clinical datasets or third party data, please ensure that the statement adheres to our policy

The ChIP-seq, RNA-seq, BL-Hi-C, QHR-4C, snRNA-seq and scRNA-seq data reported in this paper have been deposited in the Gene Expression Omnibus (GEO) database under accession code GSE214692 (https://www.ncbi.nlm.nih.gov/geo/query/acc.cgi?acc=GSE214692) and in the Genome Sequence Archive database90 in the National Genomics Data Center91 (GSA) under accession code CRA008223 (https://ngdc.cncb.ac.cn/gsa/browse/CRA008223) and HRA003128 (https://ngdc.cncb.ac.cn/gsa-human/browse/HRA003128). The dataset of this paper has been submitted to the figshare repository (https://doi.org/10.6084/m9.figshare.22762457). The referenced Allen fetal brain data is available from the European Genome Phenome Archive under accession code EGAS00001004107 (https://ega-archive.org/datasets/EGAD00001006049). The GWAS table source of autism spectrum disorder used in this study is available in the GWAS Catalog under accession code EFO_0003756 (https://www.ebi.ac.uk/gwas/efotraits/EFO_0003756). Source data are provided with this paper.

Research involving human participants, their data, or biological material

Policy information about studies with human participants or human data. See also policy information about sex, gender (identity/presentation), and sexual orientation and race, ethnicity and racism.

Reporting on sex and gender	*Use the terms sex (biological attribute) and gender (shaped by social and cultural circumstances) carefully in order to avoid confusing both terms. Indicate if findings apply to only one sex or gender; describe whether sex and gender were considered in study design; whether sex and/or gender was determined based on self-reporting or assigned and methods used.* *Provide in the source data disaggregated sex and gender data, where this information has been collected, and if consent has been obtained for sharing of individual-level data; provide overall numbers in this Reporting Summary. Please state if this information has not been collected.* *Report sex- and gender-based analyses where performed, justify reasons for lack of sex- and gender-based analysis.*
Reporting on race, ethnicity, or other socially relevant groupings	*Please specify the socially constructed or socially relevant categorization variable(s) used in your manuscript and explain why they were used. Please note that such variables should not be used as proxies for other socially constructed/relevant variables (for example, race or ethnicity should not be used as a proxy for socioeconomic status).* *Provide clear definitions of the relevant terms used, how they were provided (by the participants/respondents, the researchers, or third parties), and the method(s) used to classify people into the different categories (e.g. self-report, census or administrative data, social media data, etc.)* *Please provide details about how you controlled for confounding variables in your analyses.*
Population characteristics	*Describe the covariate-relevant population characteristics of the human research participants (e.g. age, genotypic information, past and current diagnosis and treatment categories). If you filled out the behavioural & social sciences study design questions and have nothing to add here, write "See above."*
Recruitment	*Describe how participants were recruited. Outline any potential self-selection bias or other biases that may be present and how these are likely to impact results.*
Ethics oversight	*Identify the organization(s) that approved the study protocol.*

Note that full information on the approval of the study protocol must also be provided in the manuscript.

Field-specific reporting

Please select the one below that is the best fit for your research. If you are not sure, read the appropriate sections before making your selection.

☒ Life sciences ☐ Behavioural & social sciences ☐ Ecological, evolutionary & environmental sciences

For a reference copy of the document with all sections, see nature.com/documents/nr-reporting-summary-flat.pdf

Life sciences study design

All studies must disclose on these points even when the disclosure is negative.

Sample size	In this study, mouse model and organoid model derived from human embryonic stem cells (hESCs) were used. For the mouse phenotype experiments, we typically used a sample size of more than 3 mice per group. For behavioral studies, we used a sample size of more than 8 mice per group. However, for some experiments, such as the MEA experiments, it was challenging to obtain homozygous samples from the

	same batch, resulting in a smaller sample size of n=2. For organoid differentiation, we used 2 clones for each genotype.
Data exclusions	We did not exclude data from the analysis.
Replication	Phenotypes observed are robust and were reliably reproduced at least three biological repeats. Except for adjusted ChIP-seq and single-cell sequencing data, all other sequencing data types had at least two biological replicates.
Randomization	In this study, the mice used for analyzing growth phenotypes, neurological phenotypes, behavioral performance and other experiments were randomly selected. The screening of hESC clones and organoid selection were also random.
Blinding	We adhered to the blinding process during the behavioral experiments to ensure unbiased data collection. However, for certain experiments such as sequencing, the experimental design necessitated prior knowledge of the genotype of the mice to select the appropriate samples. Despite this, we ensured that the analysis of the results was conducted objectively, and the data was interpreted without any bias.

Reporting for specific materials, systems and methods

We require information from authors about some types of materials, experimental systems and methods used in many studies. Here, indicate whether each material, system or method listed is relevant to your study. If you are not sure if a list item applies to your research, read the appropriate section before selecting a response.

Materials & experimental systems

n/a	Involved in the study
☐	☒ Antibodies
☐	☒ Eukaryotic cell lines
☒	☐ Palaeontology and archaeology
☐	☒ Animals and other organisms
☒	☐ Clinical data
☒	☐ Dual use research of concern
☒	☐ Plants

Methods

n/a	Involved in the study
☐	☒ ChIP-seq
☒	☐ Flow cytometry
☒	☐ MRI-based neuroimaging

Antibodies

Antibodies used	Primary antibodies:<br>Mouse monoclonal anti-SATB2 (Abcam, Cat# AB51502, dilution 1:200), Mouse monoclonal anti-MAP2 (Sigma, Cat# M4403, dilution 1:100), Rabbit monoclonal anti-SOX2 (Cell Signaling Technology, Cat# 23064, dilution 1:200), Rabbit polyclonal anti-βIII-tublin(TUJ1) (Abcam, Cat# AB18207, dilution 1:200) for immunofluorescence.<br>Rabbit polyclonal anti-CTCF (Active motif, Cat# 61311, dilution 1:500) for immunohistochemistry.<br>Rabbit polyclonal anti-CTCF (Active motif, Cat# 61311), Rabbit monoclonal anti-RAD21 (Abcam, Cat# ab217678), Rabbit monoclonal anti- IgG (Abcam, Cat# ab37415) 5 μg per ChIP.<br>Rabbit polyclonal anti-CTCF (Millipore, Cat# 07-729, dilution 1:1000), Mouse monoclonal Anti-CTCF (Abcam, Cat# ab37477, dilution 1:1000), Mouse monoclonal anti-β-ACTIN (Sigma-Aldrich, Cat# A2228, Lot# 0000165975, dilution 1:5000) for WB.<br>Secondary Antibody:<br>Goat Anti-Mouse IgG (H+L) HRP (KangChen, Cat# KC-MM-035, dilution 1:5000 for WB), Goat anti-Rabbit IgG (H&L) HRP (KangChen, Cat# KC-RB-035, dilution 1:5000 for WB), goat anti-rabbit IgG Alexa 594 secondary antibody (Invitrogen, Cat# A11012, dilution 1:300 for immunofluorescence), goat anti-mouse IgG Alexa 488 secondary antibody (Invitrogen, Cat# A11001, dilution 1:300 for immunofluorescence), goat anti-rabbit IgG Alexa 488 secondary antibody (Invitrogen, Cat# A11008, dilution 1:300 for immunofluorescence), goat anti-rabbit HRP (Cell Signaling Technology, Cat# 8114 for immunohistochemistry).
Validation	All antibodies used in this study are commercial which were validated by following the manufacturer's instructions.

Eukaryotic cell lines

Policy information about cell lines and Sex and Gender in Research

Cell line source(s)	HEK293T cell lines were purchased from ATCC (Cat# ATCC® CRL-11268TM).<br>H1 hESCs purchased from WiCell (Cat# WA01) were kindly provided by our collaborative colleagues in CAS Key Laboratory of Regenerative Biology, Centre for Regenerative Medicine and Health, Guangzhou Institutes of Biomedicine and Health, Chinese Academy of Sciences.
Authentication	HEK293T cell lines were derived from authenticated stock from ATCC. H1 cell lines were derived from authenticated stock from WiCell. Routine quality control was measured with microscopy morphology, and qRT-PCR was performed to test the expression of marker genes.
Mycoplasma contamination	All cell lines were confirmed negative for mycoplasma contamination.

Commonly misidentified lines (See ICLAC register)	There were no commonly misidentified cell lines were used in the study.

Animals and other research organisms

Policy information about studies involving animals; ARRIVE guidelines recommended for reporting animal research, and Sex and Gender in Research

Laboratory animals	The mice (both wild-type and CTCF mutation) used in this study were C57BL/6N mice which were generated and purchased from Cyagen Biosciences. The mice used in this study were E18.5 and 2-3 month-old with no sex preference.
Wild animals	This study did not involve wild animals.
Reporting on sex	In this study, our previous observations of the growth phenotype of the mice in this study showed no sex preference.
Field-collected samples	This study did not involve samples collected from the field.
Ethics oversight	The animal experiments were performed in accordance with the Guidelines for the Care and Use of Laboratory Animals of the National Institutes of Health. The protocol was approved by the Committee on the Ethics of Animal Experiments of Guangzhou Institutes of Biomedicine and Health, Chinese Academy of Sciences.

Note that full information on the approval of the study protocol must also be provided in the manuscript.

Plants

Seed stocks	*Report on the source of all seed stocks or other plant material used. If applicable, state the seed stock centre and catalogue number. If plant specimens were collected from the field, describe the collection location, date and sampling procedures.*
Novel plant genotypes	*Describe the methods by which all novel plant genotypes were produced. This includes those generated by transgenic approaches, gene editing, chemical/radiation-based mutagenesis and hybridization. For transgenic lines, describe the transformation method, the number of independent lines analyzed and the generation upon which experiments were performed. For gene-edited lines, describe the editor used, the endogenous sequence targeted for editing, the targeting guide RNA sequence (if applicable) and how the editor was applied.*
Authentication	*Describe any authentication procedures for each seed stock used or novel genotype generated. Describe any experiments used to assess the effect of a mutation and, where applicable, how potential secondary effects (e.g. second site T-DNA insertions, mosiacism, off-target gene editing) were examined.*

ChIP-seq

Data deposition

- [x] Confirm that both raw and final processed data have been deposited in a public database such as GEO.
- [ ] Confirm that you have deposited or provided access to graph files (e.g. BED files) for the called peaks.

Data access links *May remain private before publication.*	ChIP-seq data reported in this paper have been deposited in the Genome Sequence Archive in National Genomics Data Center, China National Center for Bioinformation/Beijing Institute of Genomics, Chinese Academy of Sciences (GSA: CRA008223 and HRA003128) and in the Gene Expression Omnibus (GEO) database (GSE214692) in NCBI.
Files in database submission	Brain_CTCF_homo_R1.fq.gz<br>Brain_CTCF_wt_R1.fq.gz<br>Heart_CTCF_homo_R1.fq.gz<br>Heart_CTCF_wt_R1.fq.gz<br>Lung_CTCF_homo_R1.fq.gz<br>Lung_CTCF_wt_R1.fq.gz<br>Brain_CTCF_homo_R2.fq.gz<br>Brain_CTCF_wt_R2.fq.gz<br>Heart_CTCF_homo_R2.fq.gz<br>Heart_CTCF_wt_R2.fq.gz<br>Lung_CTCF_homo_R2.fq.gz<br>Lung_CTCF_wt_R2.fq.gz<br>Input_Brain_homo_R1.fq.gz<br>Input_Brain_homo_R2.fq.gz<br>Input_Brain_wt_R1.fq.gz<br>Input_Brain_wt_R2.fq.gz<br>Input_Lung_homo_R1.fq.gz<br>Input_Lung_homo_R2.fq.gz<br>Input_Lung_wt_R1.fq.gz<br>Input_Lung_wt_R2.fq.gz

Input__Heart_homo_R1.fq.gz
Input__Heart_homo_R2.fq.gz
Input__Heart_wt_R1.fq.gz
Input__Heart_wt_R2.fq.gz

Genome browser session (e.g. UCSC)	BW files were visualized in IGV and the reference genome assembly was Mouse GRCm38/mm10.

Methodology

Replicates	The adjusted ChIP experiments were performed twice by adding an equal amount of HEK293T chromatin for normalization and only one adjusted experiment was used for subsequent analysis.
Sequencing depth	All above sequencing data were at least 30 M reads; unique reads were at least 10 M. Detailed mapping statistic and peak calling information for ChIP-seq data were described in Supplementary Data 6.
Antibodies	Rabbit polyclonal anti-CTCF (Active motif, Cat# 61311)
Peak calling parameters	Reads mapped to the mouse or human genome were separated and then subjected to peak calling using macs2 (version 2.2.7.1) with default options.
Data quality	Raw reads were trimmed to remove adaptors using Trim Galore and then mapped to the mouse (mm10) and human (hg38) mixed genomes using bowtie2 (version 2.2.5) with the following parameters: --very-sensitive --end-to-end --no-unal --no-mixed --no-discordant. Only uniquely mapped reads with MAPQ > 30 were retained.
Software	Trim Galore, bowtie2, macs2, deepTools2
